# Text Messaging in Cancer-Supportive Care: A Systematic Review

**DOI:** 10.3390/cancers13143542

**Published:** 2021-07-15

**Authors:** Don Thiwanka Wijeratne, Meghan Bowman, Isobel Sharpe, Siddhartha Srivastava, Matthew Jalink, Bishal Gyawali

**Affiliations:** 1 Department of Medicine, Queen’s University, Kingston, ON K7L 3N6, Canada; dtdw@queensu.ca (D.T.W.); srivastava.s@queensu.ca (S.S.); matt.jalink@queensu.ca (M.J.); 2 Department of Public Health Sciences, Queen’s University, Kingston, ON K7L 3N6, Canada; 19melb@queensu.ca (M.B.); isobel.sharpe@queensu.ca (I.S.); 3 Department of Oncology, Queen’s University, Kingston, ON K7L 3N6, Canada

**Keywords:** cancer, text-based communication, text messaging, mHealth, supportive care, systematic review

## Abstract

**Simple Summary:**

As the number of patients with cancer continues to rise globally, developing methods to support these patients is important for providing high-quality care. The aim of our systematic review is to describe and examine the use of text-based communications in cancer supportive care. We identified 18 studies suitable for inclusion in our review. Overall, patients were satisfied with using text-based communication in their cancer care. Results were inconsistent within other outcome categories; however, specific interventions yielded some positive results such as the use of reminders for promoting medication adherence. While the results of this review should not be considered conclusive, it illustrates that further research on the use of text-based communications in cancer-supportive care is warranted.

**Abstract:**

The global cancer disease burden is substantial, resulting in increased economic and clinical strain on our healthcare systems. A proposed solution is text-based communication, which can be used for cancer-supportive care. We conducted a systematic review to synthesize and describe the use of text-based communications for cancer-supportive care. Our population of interest included adult patients with cancer. A total of 18 studies were included in the review: 9 RCTs and 9 non-randomized interventional/observational studies. Patients were largely satisfied with text-based communication during their cancer care. Compared to controls, results for other outcomes including symptoms and quality of life were largely mixed; however, no harms were observed. Furthermore, positive outcomes were seen for specific interventions, such as text message medication reminders. These findings should be considered with caution due to the considerable heterogeneity observed between studies regarding their design and reported outcomes and the high risk of bias associated with 6/18 studies. Overall, this review suggests that text-based communication may be a complementary tool for cancer-supportive care; however, more research is needed to examine the feasibility of implementation and use.

## 1. Introduction

As the global cancer incidence continues to rise, so has the need for supportive services. Acting as an integral component of cancer care, supportive care aims to provide relief from side effects and aid in care management [1]. Patients with advanced cancer experience high symptom burden, which is associated with prolonged hospital stays and unplanned hospital readmissions [2]. Supportive care services for patients with cancer is a substantial healthcare cost [3]. The annual excess economic burden of cancer survivorship ranges from $4427 USD to $16,441 USD per survivor [4]. Given the considerable clinical and economic burden of caring for those with cancer, it is important to develop cost-effective and efficient supportive care strategies.

Digital health interventions facilitate independence and provide reassurance to patients with cancer [5]. Literature suggests that text-based interventions may be a promising alternative or addition to more traditional telehealth methods, such as phone call and internet-based interventions [6]. They have been shown to facilitate patient–provider communication, enhance adherence via medication reminders [7], educate and motivate patients in the self-management of their care, and simplify patient data collection [8,9]. Specifically, among patients with cancer, text-based communication has been found to improve patient communication and self-management of their care [10].

Text-based communication can complement other internet-based communication methods [11]. Employing internet-based communication as a sole method of communication may exacerbate quality of care inequities, as many patients with cancer have limited Internet access [12]. The World Internet Project, a collaborative research program that studies the impact of digital technology, reported wide disparities in Internet usage based on income levels in almost all countries [13].

The use of text-based communication is a relatively new mode of supporting cancer patients. While the literature suggests these interventions may be cost-effective and aid in patient-centered care, much of the available evidence is not disease-specific [9,14,15]. Previous reviews have examined text-based communication as a component of or under the umbrella of digital health or mHealth [8,16,17], or have examined text-based communication interventions in patients with chronic disease as a whole [15,18,19]. Reviews investigating the effects of text-message interventions specifically among patients with cancer are limited, and a full scope of patient-related outcomes has yet to be investigated. Therefore, we conducted a systematic review aimed at identifying and describing the use of text-based communications to provide supportive care for cancer patients. 

## 2. Materials and Methods

We conducted a systematic review in accordance with the Preferred Reporting Items for Systematic Reviews and Meta-Analyses (PRISMA) checklist [20].

### 2.1. Eligibility Criteria

Our review included primary research studies (clinical trials and observational studies; all study designs) with an available full text and published in the English language. Conference abstracts (i.e., no available full text), reviews, case studies, case series, and commentaries/opinions were excluded. Our population of interest was adult patients (age ≥18 years) with diagnosed cancer. Our intervention of interest was use of text-based communication to assist in cancer-supportive care [1]. Studies that included text-based communication as part of a multi-modal intervention or those requiring internet were not included as we were unable to discern the individual effects of text-based communication. The primary outcome was cancer symptom control. Secondary outcomes included quality of life (defined as a multidimensional phenomenon made up of multiple domains including mental and physical health, physical activity, diet, etc., [21]), feasibility (e.g., adherence, implementation), patient satisfaction (e.g., patient opinions of the intervention including overall satisfaction, helpfulness, usefulness), and barriers to use. Both quantitative and qualitative outcomes were included. Table S1 contains detailed inclusion and exclusion criteria.

### 2.2. Search Strategy

Electronic searches were performed in MEDLINE, EMBASE, and CINAHL from January 2015 to November 2020. A previous study [22] surveyed literature up to 2015 on mobile technologies for cancer supportive care. Since the technology and utility of text-based communication has substantially changed in recent years, we chose to evaluate the studies published since 2015 making our results applicable to the current context. Table S2 contains the full search strategy and number of results corresponding to each database.

### 2.3. Data Extraction and Outcome Measures

The Covidence platform [23] was used to organize and screen all the studies identified by the search. Search results from all three databases were aggregated and duplicates were removed. Two independent reviewers (MB, IS) screened the studies at the title/abstract level for inclusion. The remaining studies were then assessed by the two reviewers for eligibility at the full-text level. Any studies deemed ineligible were excluded based on the following hierarchy: study design, population, exposure/intervention, and outcomes. Conflicts between the reviewers were resolved through mutual discussion and agreement, or, if no consensus was reached, by a third arbitrator (DTW/BG).

Upon finalizing the included studies, the two reviewers (MB, IS) extracted relevant information using a predeveloped Microsoft Excel form. Detailed information was collected on the study design, location, population, baseline characteristics, intervention, control, and outcomes.

### 2.4. Bias Assessment

Bias assessments were performed on each study by two independent reviewers (MB, IS). The Cochrane Risk of Bias Tool [24], a 7-item tool that judges biases related to selection, performance, detection, attrition, reporting, and others, was used to assess the randomized trials. Each of the 7 items was scored on a 2-point scale, where a score of 2 represents low risk of bias, 1 represents an unclear risk of bias, and 0 represents high risk of bias. The individual item scores were then summed; a total score of 8 or higher was considered low risk of bias and a score below 8 was considered high risk of bias. The Risk Of Bias In Non-randomized Studies-of Interventions (ROBINS-I) tool [25] was used to assess the non-randomized interventional/observational studies. This is a 7-item tool that judges biases related to confounding, selection, intervention classification and adherence, missing data, and outcome measurement and reporting. Biases corresponding to each of the 7 items were rated as low, moderate, severe, critical, or no information and an overall score was also given.

## 3. Results

### 3.1. Overview

**Figure 1:**
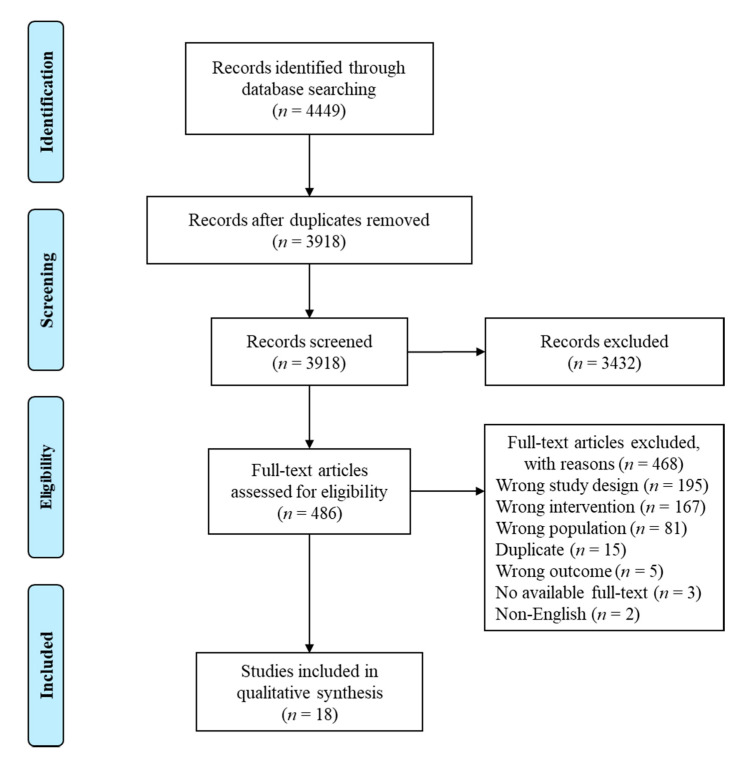
PRISMA flow diagram.

The electronic database search identified a total of 4449 studies, out of which 18 studies involving 5821 patients were included in the final qualitative synthesis (Figure 1). Of the included studies, 9 (50%) were randomized trials [26,27,28,29,30,31,32,33,34] and 9 (50%) were non-randomized interventional/observational studies [11,35,36,37,38,39,40,41,42] (Table 1, Table S3). Sixteen of 18 studies (89%) were conducted in high-income countries (HICs) [11,26,27,28,29,31,32,33,34,35,36,37,38,40,41,42], the majority (*n =* 11) in the United States [11,26,28,29,33,34,35,36,38,40,41]. Two studies (11%) were conducted in low- and middle-income countries (LMICs), both in Brazil [30,39].

Ten studies included patients with any cancer [26,27,30,32,33,34,36,39,41,42], while the remaining eight focused on specific cancer types (breast *n =* 4 [11,29,31,38], lung *n =* 2 [35,37], endometrial *n =* 1 [28], chronic myeloid leukemia *n =* 1 [40]) as seen in Table 1. One study included patients with late-stage cancer only (stage III–IV lung) [35] and three studies included patients with early stage cancer only [11,29,38], while the remainder did not use staging as part of their inclusion criteria. Further, 13 studies focused on patients in periods of active treatment [11,29,30,31,32,33,34,36,37,38,39,40,41], while 4 studies focused on patients during their survivorship phase [26,27,28,42] and 1 did not specify [35]. The total sample sizes at baseline ranged from 14 [39] to 3429 participants [41].

Sixteen studies assessed text-based communication interventions used solely for supportive care purposes, such as medication adherence, appointment reminders, weight loss, physical activity motivation, and self-management education [11,26,27,28,29,30,31,32,33,34,35,36,39,40,41,42]. The remaining two studies used text-based communication for supportive care as well as for symptom monitoring (i.e., tracking symptoms experienced by patients) [37,38]. The most common message frequency was daily (*n =* 7) [11,30,33,34,38,39,41], followed by weekly (*n =* 3) [31,32,36], multiple times per week (*n =* 3) [27,29,42], and multiple times per day (*n =* 3) [28,35,37]. Two studies had no set message frequency [26,40]. Text-based communication was initiated by the provider in 16 studies [11,27,28,29,30,31,32,33,34,35,36,38,39,40,41,42] and by the patient in two studies [26,37]. Thirty-nine percent (*n =* 7) of studies sent messages that were personalized to the individual patient [26,27,28,35,37,40,41], while the remainder used non-personalized messages [11,29,30,31,32,33,34,36,38,39,42]. Eleven studies used unidirectional text-based communication (provider to patient) [11,29,30,31,32,33,34,35,39,41,42], six used bidirectional communication (provider to patient and patient to provider) [26,27,28,37,38,40], and the remainder used text-based communication to initiate an additional form of communication (phone call, mobile app, patient portal, etc.,) [36]. Of the eight studies that reported the language used for text messaging, four [26,28,33,34] used English, two [30,39] used Portuguese, one [32] used French, and one [31] had the option of either English, Mandarin, or Malay. Study follow-up duration ranged from 1 month [35,36,37,39,42] to 3 years [29]. The following sections discuss study findings within five main outcome categories: patient satisfaction, barriers to use, symptoms, QoL, and feasibility.

### 3.2. Risk of Bias

Seven of the nine randomized trials were classified as low risk of bias (LRB), while the remaining two were high risk of bias (HRB) (Figure S1, Table S4). Of the nine non-randomized interventional/observational studies, six reported a serious risk of bias (some important problems exist), two reported not enough information to judge level of bias, and the final study reported moderate risk of bias (sound for a non-randomized study, but not comparable to a well-performed randomized trial) (Figure S2, Table S5). Overall, a large portion of the included studies were at a high risk of bias and should be considered cautiously. To provide context for readers interpreting our findings, we have denoted the level of bias as either low risk (LRB) or high risk (HRB) throughout the subsequent results sections.

### 3.3. Patient Satisfaction

Twelve studies (67%) reported outcomes related to patient satisfaction (Table 2). Overall, patient satisfaction with the text-based interventions was high. For example, Bade et al. [35] reported that 92% of patients found a twice-daily motivational text communication service helpful for improving their physical activity (HRB). In a pilot study of text messages for self-care and emotional support (sent using the cHEmotHErAPP), Rico et al. [39] reported that all patients felt they could better cope with their cancer treatment through feelings of increased confidence and knowledge (LRB). Similarly, a two-armed, prospective, single-center RCT of the same intervention reported a patient satisfaction rate of 100% in the intervention group, where 72% were very satisfied and 28% were satisfied (HRB) [30]. Krok-Schoen and colleagues [11] conducted a pre-post study of daily text message reminders to improve hormone therapy adherence among breast cancer patients and found that 97% had a positive experience (HRB).

### 3.4. Barriers to the Use of Text-Based Communication

Seven studies (39%) reported barriers to the use of text-based communication (Table 2). Three of those studies [11,27,38] reported barriers to text-based communication adherence, which included scheduling conflicts, forgetfulness, feeling unwell, not liking the language used in the messages, and not feeling well enough to continue without the messages [11,27]. The study by Mougalian and colleagues [38] was the only study to document cost-related outcomes, where over 70% of patients had no financial impact associated with the text-based communications (HRB). Three studies [33,34,37] identified technological barriers to completing the intervention, and overall reported few technological problems. For example, Spoelstra et al. [34] found that just 5.3% of patients encountered problems with the medication adherence text messaging system in their two-armed, prospective, multi-center RCT (LRB). Lastly, two studies [37,42] reported barriers to study recruitment, which included poor health status, lack of familiarity/confidence with the technology, and perceived lack of need for additional supportive interventions (HRB, HRB respectively).

### 3.5. Symptom Outcomes

Six studies (33%) examined symptoms (Table 3). Overall, results for composite symptom scores were mixed while symptom-specific results were not significantly different between study groups. In terms of composite scores, Rico et al. [30] found that the total number of side effects from cycle one of chemotherapy was significantly lower in the text-based communication group compared to those receiving standard care (number of patients experiencing 4–14 side effects in intervention (I) vs. control (C) groups: *n =* 28 vs. 42, *p =* 0.05, HRB), but this was not seen in the following two chemotherapy cycles (cycle 2: *p =* 0.4, cycle 3: *p =* 0.4). Conversely, Krok-Schoen et al. [11] found no significant difference in mean Breast Cancer Prevention Trial Symptom Checklist score post treatment with text-based communication vs. baseline (mean difference (MD) (post-baseline) 0.04, 95%CI -0.06 to 0.14, *p =* 0.412, HRB). Despite the heterogeneity between studies, positive results were found in specific settings such as Spoelstra et al. [33]. This study [33] was a two-armed, prospective, multi-center RCT examining a population of patients with various forms of cancer and newly prescribed oral anticancer medication. A significant reduction in mean total number of symptoms was reported among those receiving daily medication adherence text messages compared to those who did not (I vs. C mean 3.9 SD 0.5 vs. 5. SD 0.46, *p =* 0.04, LRB).

Among symptom-specific results, Krok-Schoen et al. [11] found no significant change in mean Brief Pain Inventory Score (MD (I–C) 0.8, 95% CI −0.4 to 2.02, *p* = 0.2) or mean Fatigue Symptom Inventory (MD (I–C) −0.3, 95% CI −0.8 to 0.3, *p* = 0.3) from post-treatment vs. baseline (HRB).

### 3.6. Quality of Life Outcomes

A total of ten studies (56%) examined the QoL outcomes (Table 3). Of these, six studies [11,27,28,32,35,37] examined the physical health outcomes including step counts, physical activity change, physical fatigue, and self-care change. Of the two studies that reported step counts one found no consistent change in steps in the text-based communication vs.no text communication groups over an 8-week period (HRB) [32], while the other found that mean daily steps significantly increased for both the text-based communication and weekly phone call groups over a 3 week period compared to controls (effect sizes 0.02 and 0.05, respectively, HRB) [35]. Physical activity change was reported by two studies and mixed results were found. In their single-center prospective RCT, Gomersall et al. [27] reported an increase in physical activity among those receiving text-based communication versus those undergoing a 4-week exercise rehabilitation without texts (mean time light stepping, change score MD (I–C) 6.9, 95% CI 0.8, 12.9, *p* = 0.03, LRB). Physical component scores from various questionnaires (Functional Assessment of Cancer Therapy-Lung (FACT-L), Short Form Health Survey (SF-8), Quality of Life Questionnaire (QLQ-30), Patient-Reported Outcomes Measurement Information System (PROMIS)) reported mixed results. Krok-Schoen et al. [11] found that physical health, as reported by the SF-8, was better at post-treatment vs.baseline, though this difference was not significant (MD (I–C) 0.95, 95% CI −1.71 to 3.61, *p* = 0.473, HRB). In their single-center RCT, Villaron et al. [32] reported significant results for the physical capacity of the QLQ-30, finding increased capacity among those receiving text-based communication vs.no text messages. However, their results were significant only within 7 weeks of follow-up (I vs. C: mean 88.3 SD 13.5 vs. 75.5 SD 18.9, *p* < 0.01, HRB).

Seven [11,32,33,34,36,37,42] studies looked at an array of mental health outcomes including depression, anxiety, motivation, and emotional well-being (Table 3). Depression was the most highly reported outcome (33% [11,33,34,36,37,42] studies), and results were varied. In their multi-center RCTs, Spoelstra et al. [34] and Spoelstra et al. [33] both found no significant difference in PROMIS depression scores among those receiving text-based communication vs.standard care (*p* = 0.8, *p* = 0.9, respectively, LRB). Krok-Schoen and colleagues [11] found those who received texts to prompt medication adherence had lower Center of Epidemiology Study-Depression (CESD-D) score compared to baseline scores (MD −1.2, 95% CI −3.5, 1.01, *p* = 0.3, HRB). Similar results were found with anxiety outcomes; Wells et al. [42] reported a trend for greater improvement in anxiety among those receiving text-based communication to support mindfulness-based cognitive therapy (MBCT) (*p* < 0.001, HRB), however this did not significantly differ from those receiving MBCT without the additional supportive texts.

Among patients undergoing outpatient chemotherapy or systemic treatment for various forms of cancer, Villaron et al. [32]’s RCT found significantly lower decline in motivation (I vs. C week 8 mean 7.88 SD 3.74 vs. 10.73 SD 3.87 *p* < 0.05), activity (I vs. C week 8 mean 8.24 SD 4.09 vs. 11.82 SD 4.16 *p* < 0.01), and both mental (I vs. C week 8 mean 6.94 SD 3.77 vs. 9.95 SD 4.19 *p* < 0.05), and physical functioning (I vs. C week 8 mean 9.76 SD 4.63 vs. 12.27 SD 4.63 *p* < 0.05) among those receiving text-based communication that provided motivation to encourage physical activity vs. those receiving no text messages (HRB).

### 3.7. Feasibility and Implementation Outcomes

Sixteen studies (89%) examined feasibility or implementation outcomes (Table 4). A variety of feasibility outcomes were reported, including adherence to the intervention and medications, number of text messages sent, text-message response rate, and odds of completing the intervention. Seven studies [11,29,31,34,38,40] reported adherence to prescribed medications or therapy, with mixed results. Three studies reported an adherence rate > 50% [31,38,40] (HRB, HRB, LRB, respectively) among those receiving text-based communication. Conversely, one study found adherence of <50% [29] among those receiving text-based communication (LRB). Those that examined the medication adherence between study groups found mixed results. One study [11] found control groups not receiving texts to have higher adherence scores compared to the text-message intervention group (*p* < 0.05, HRB) while two studies [31,40] found that the text-based communication groups had higher adherence levels (*p* < 0.05, HRB).

On a systems level, number of text messages sent was the most ubiquitous feasibility outcome [27,30,33,38]. Number of text messages sent ranged from 52 messages over 3 cycles of chemotherapy (LRB) [30] to 21 messages sent per week (HRB) [38]. Outcomes such as text message response rates and number of alerts generated were reported by fewer studies. Text message response rate ranged from 40% (HRB) [40] to 86.1% (HRB) [38] among studies in which it was reported [27,38,40].

## 4. Discussion

In this systematic review of text-based communications in cancer-supportive care, we find that text-based communication tools are a potentially feasible intervention that tends to improve patient satisfaction, with heterogenous but no detrimental effect on symptom or QoL outcomes. However, the majority of studies included in this review were judged to be at a high risk of bias, suggesting that our results should be interpreted with caution. These findings help establish the role of text-based communication in cancer-supportive care and provide a benchmark for implementation in cancer-care facilities. They also highlight the need for future, well-designed studies.

Text-based communication is a pragmatic virtual care tool, enabling real-time bidirectional communication between provider and patient at a low cost and in an accessible format [43,44]. Amid heterogeneous results, we identified potential support for the use of text-based interventions within specific settings. For example, providing a specific call to action (e.g., for medication adherence or appointment attendance) via text-based communication may be successful in producing the desired behavior change [31,40,41,42], thereby potentially translating to improved clinical outcomes [33,34]. Similar text-driven behavioral changes have been seen among other chronic disease populations. A meta-analysis looking specifically at text-messaging for medication adherence in chronic diseases reported a significant improvement in medication adherence compared to those without text-messages [7]. Our findings also suggest that when interventions facilitate self-management, improvements in fatigue and QoL outcomes can occur. This may be due to the nature of self-management interventions, which usually include an information-based component aimed at building self-efficacy, known as a key causal mechanism for behavior change and improved health outcomes [45]. Notably, many of these positive outcomes were seen in the RCTs as opposed to the non-RCTs, indicating a higher quality of evidence in support of text-based communication feasibility. 

Our review suggests that text-based communication interventions were consistently associated with high levels of patient satisfaction and produced no harmful effects. The majority of studies reported positive patient satisfaction outcomes such as improved self-management skills, increased confidence and knowledge, and general feelings of positivity. Further, patients reported that text-based communications were helpful and easy-to-understand. These findings align with the existing research; a recent systematic review of telehealth interventions found that patient satisfaction outcomes were generally positive [46]. Notably, only one included study assessed intervention costs, showing no added cost to the patient with text-based communication [38]. This was unsurprising, given that the literature is sparse regarding cost-effectiveness of text-based interventions [44,47]. While more research is needed, high patient satisfaction suggests that text-based interventions are well-accepted and may help to reduce some of the patient-perceived barriers to care. 

We observed an overall lack of evidence for understanding the real-world effectiveness of text-based communication. Although 89% of included studies reported on feasibility, the included outcomes were highly heterogeneous and few assessed the practicality of implementing this technology. Thus, future studies should utilize standardized methods for study design and outcome reporting, facilitating inter-study comparison. Researchers should consider the use of the Research, Effectiveness, Adoption, Implementation, and Maintenance (RE-AIM) framework to identify meaningful study outcomes for determining the real-world success of text-based interventions [48]. When studying text-based communications, it is important to understand factors affecting their uptake. Therefore, we also propose the use of effectiveness-implementation hybrid study designs, which facilitate timely adoption of a given clinical intervention through the simultaneous study of both its effectiveness and uptake [49]. Moving forward, these tools will help guide researchers in identifying meaningful outcomes for studying text-based communications in cancer-supportive care.

Our review identified a clear lack of evidence from LMICs. Of the 18 included studies, only 2 (11%) took place in a single LMIC (Brazil, an upper-middle-income country). Similarly, in their review of mobile technologies in cancer care, Nasi and colleagues [22] identified a total of 106 studies, of which only 13 (12%) focused on LMICs. Comparable trends also exist in the broader cancer literature. A retrospective analysis of all phase 3 treatment studies published from 2014–2017 (*n* = 694 RCTs) found that just 8% were led by LMIC investigators [50]. LMICs experience high cancer incidence as well as disproportionately high mortality [51]. For example, in 2020 Asia reported 49.3% of global cancer cases and a disproportionate 58.3% of deaths [52]. As of 2015, approximately 8 in 10 individuals in developing countries owned a mobile phone [53]. Thus, future research efforts should focus on testing the feasibility, acceptability, and effectiveness of text-based communication in LMICs.

In the context of the pandemic, the use of telemedicine is gaining global popularity globally including in LMICs [54]. This study captured both high-quality RCTs and real-world observational studies. Although none of the studies included in our review were conducted in the context of the pandemic, this review will provide a benchmark for better integrating supportive care for cancer patients in HICs and LMICs using a text-based communication both during and after the pandemic.

### Strengths and Limitations

Our review captured both interventional and observational study designs. While interventional studies provide a higher standard of evidence, observational studies are useful for understanding the long-term and real-world outcomes. Additionally, our review focused on studies published in the past 5 years making our results timely and topical. We also performed a bias assessment of the included studies using validated risk of bias tools [24,25]. This allowed us to identify those studies at a high risk of bias and interpret their findings accordingly. Lastly, the potential for selection bias was reduced due to a rigorous study screening process. 

A limitation of this review was the substantial heterogeneity observed between studies regarding their population of interest, intervention design, outcome reporting, and follow-up duration. This heterogeneity may be due, in part, to the variety of phases in the healthcare process captured by this review. As demonstrated by Nasi et al. [22], text-based interventions can be employed at any phase of cancer care. Our review focused on the treatment and follow-up periods, two phases that tend to highlight different outcome types. Methodological standardization using tools such as the effectiveness-implementation study design and RE-AIM framework may help improve comparability of future works. In addition, several included studies were likely underpowered, with a limited ability to detect differences between treatment groups. Six non-randomized interventional studies were identified serious risk of bias and two RCTs were identified as high risk of bias. Including outcomes reported by these studies regarding the feasibility and effectiveness of text-based communications may have prevented accurate conclusions from being drawn. As most studies included had short follow-up periods, the findings of this review cannot speak for long-term effects of text-based communication. Additionally, the needs of patients with chronic diseases vary greatly, limiting to the generalizability of these results beyond patients with cancer. Lastly, for feasibility reasons our search was restricted to studies written in the English language and we did not conduct a search for grey literature. This may have limited our search and excluded relevant studies from inclusion.

## 5. Conclusions

In conclusion, text-based intervention in cancer-supportive care trends toward high levels of patient satisfaction, suggesting that this may be an acceptable tool for augmenting supportive care. Although findings were inconsistent regarding the effects of text-based communications on patient symptoms, QoL, and feasibility/implementation, we observed the potential success of text-based communication within specific settings. More research is needed to identify what is needed to achieve a successful text-based intervention. Given its low costs and more equitable coverage, text-based communication has the potential to improve global cancer care.

## Figures and Tables

**Table 1:** Study characteristics (*n =* 18 studies).

Author, Year (Country)	Study Design	Study Population, Sample Size (I vs. C)	Intervention vs. Control Description	Follow-Up
Randomized Trials				
Casillas, 2019 (United States) [26]	RCT (three-armed, parallel, prospective, single-center)	Adolescent and young adult childhood cancer survivors, 28 vs. 25 vs. 25	All groups received an education booklet and identified 3 survivorship goals. Text message group: two-way texting system that supported engagement and provided resources for achieving goals vs. Peer navigation group: telephone calls to discuss goals vs. Control group: encouraged to seek answers to questions regarding educational material	2 mos
Gomersall, 2019 (Australia) [27]	RCT (two-armed, parallel, prospective, single-center)	Cancer patients at least one month post-surgery, 18 vs. 18	4 wk exercise rehabilitation program + 12 wk tailored text messages designed to improve whole-of-day activity vs. 4 wk exercise rehabilitation program	3 mos
Haggerty, 2017 (United States) [28]	RCT (three-armed, parallel, prospective, multi-center)	Women with a history of endometrial cancer, BMI 30+, no current or planned treatments, 13 vs. 14 vs. 15	Text message group: 3–5 daily personalized interactive text messages (feedback, support, strategies for behavioral change regarding weight loss) vs. Telemonitoring group: weekly/biweekly telephone counselling vs. Control group: paper handouts on healthy eating and exercise	6 mos
Hershman, 2020 (United States) [29]	RCT (two-armed, parallel, prospective, multi-center)	Post-menopausal women with breast cancer (stage I–III) taking a third-generation aromatase inhibitor, 348 vs. 354	Twice-weekly educational text messages focusing on barriers to medication adherence vs. no text messaging	3 yrs
Rico, 2020 (Brazil) [30]	RCT (two-armed, parallel, prospective, single-center)	Outpatients undergoing chemotherapy, 59 vs. 59	Daily text messages on prevention of side effects and emotional support, sent automatically in conjunction with cHEmotHErApp vs. standard care	10 mos
Spoelstra, 2016 (United States) [34]	RCT (two-armed, parallel, prospective, multi-center)	Patients newly prescribed OA, 49 vs. 26	Daily medication adherence text messages (based on social cognitive theory, 6 used on a rotating basis) vs. standard care	9 wks
Spoelstra, 2015 (United States) [33]	RCT (two-armed, parallel, prospective, multi-center)	Patients newly prescribed OA, 40 vs. 40	Daily medication adherence text messages (based on social cognitive theory, 6 used on a rotating basis) + weekly symptom management text messages vs. standard care	9 wks
Tan, 2020 (Singapore) [31]	RCT (two-armed, parallel, prospective, multi-center)	Breast cancer patients prescribed AET for at least one year, 123 vs. 121	Weekly text message reminders to take anti-cancer medication vs. standard care	1 yr
Villaron, 2018 (France) [32]	RCT (two-armed, parallel, prospective, single-center)	Outpatients undergoing chemotherapy, 21 vs. 22	Motivational text messages sent at the beginning of each week + physical activity recommendation guide vs. no text messages or recommendation guide	2 mos
Non-Randomized Interventional/Observational Studies				
Bade, 2018 (United States) [35]	Comparative	Advanced-stage (III or IV) lung cancer before, during or after treatment, 15 vs. 29	Twice-daily personalized text messages regarding activity goals (weekly activity goal, current step count, and motivational statements) for 12 wks vs. weekly phone calls to discuss activity goals	1 mos
Chow, 2019 (United States) [36]	Single arm observational	Receiving active cancer treatment (chemotherapy), 52	Text message invitation to complete a web-based distress screener one per week for 4 wks (no control group)	1 mos
Krok-Schoen, 2019 (United States) [11]	Pre-post	Post-menopausal women with breast cancer (stage 0–III) receiving hormone therapy for the first time, 39	Daily text message reminders to take hormone therapy medication + weekly text message to prompt completion of medication adherence survey within a mobile app vs. same sample at baseline	3 mos
Maguire, 2015 (United Kingdom) [37]	Mixed methods	Lung cancer patients receiving thoracic radiotherapy, 16	Completed daily symptom questionnaires, data sent in real time to a central study server and an integrated risk model analyzed and reported symptoms. The server then generated alerts to a pager held by a health professional at the clinic (no control group)	≥1 mos
Mougalian, 2017 (United States) [38]	Pilot	HR-positive breast cancer patients (stage I–III) recommended adjuvant hormonal therapy (follow-up) 100 vs. 100	Interactive daily medication reminders, weekly AE questions, and monthly texts regarding barriers to adherence (any alerts generated were forwarded to the clinical team) vs. standard care (set of historical controls using medical records)	3 mos
Rico, 2017 (Brazil) [39]	Single arm Pilot	Outpatients undergoing chemotherapy, 14	Daily text messages promoting self-care and emotional support, sent automatically in conjunction with cHEmotHErApp (no control group)	1 mos
Sawicki, 2019 (United States) [40]	Retrospective cohort	Patients initiated on TKI therapy (follow-up), 279 vs. 279	Interactive text messages on lab testing, adherence to prescribed therapy, symptoms and side effects, and condition-specific management guidance (with links to request a consultation) + non-interactive medication refill reminders vs. non-interactive medication refill reminders	1 yr
Tan, 2019 (United States) [41]	Retrospective cohort	Cancer patients undergoing radiation therapy, 668 vs. 2761	Text messaging platform connected to medical records, sent appointment reminders 2 hours prior vs. no reminders	7 mos
Wells, 2020 (United Kingdom) [42]	Mixed methods	Cancer patients receiving treatment and having mild- moderate clinical anxiety and/or depressive symptoms, 30 vs. 21	MBCT intervention focused on mindfulness skills + text message reminders (reminded patients of home practice, info from previous sessions, upcoming sessions) vs. MBCT intervention (opted out of text messaging service)	1 mos

Abbreviations: adjuvant endocrine therapy (AET), adverse events (AE), body mass index (BMI), control group (C), hormone receptor positive (HR-positive), intervention group (I), mindfulness-based cognitive therapy (MBCT), month(s) (mos), oral anticancer (OA), randomized controlled trial (RCT), tyrosine kinase inhibitor (TKI), week (wk), year (yr).

**Table 2:** Patient satisfaction (*n =* 12 studies) and barrier (*n =* 7 studies) outcomes.

Author, Year	Patient Satisfaction Outcomes ^a^	Barrier Outcomes ^b^
Randomized Trials		
Gomersall, 2019 [27]	Mean satisfaction with text messages was 4.1 SD 1.1 (*n =* 17, scores could range from 1–5)	All intervention patients attended both tailoring sessions and received text messages for the first 4 weeks of the program, however 4 participants opted out from receiving texts for the last 8 weeks (reasons included: *n =* 1 sufficiently self-motivated to continue without texts, *n =* 1 not finding texts useful, *n =* 1 overseas travel, *n =* 1 not liking the directive language of the texts)
Rico, 2020 [30]	72.1% reported being very satisfied	
Spoelstra, 2016 [34]	92% reported satisfaction (very much/highly satisfied)	5.3% encountered problems with the text message system
Spoelstra, 2015 [33]	All were somewhat (*n =* 2) or highly (*n =* 35) satisfied with their participation in the study	7/37 encountered a problem with automated voice recordings, 1/36 encountered a problem with texts
Tan, 2020 [31]	Overall most patients agreed that text messages were easy to understand (99.2%)	
Non-Randomized Interventional/Observational Studies		
Bade, 2018 [35]	92% of patients found intervention helpful (out of *n =* 13)	
Chow, 2019 [36]	Mean USE scale score was 6.9/7 for ease of use, 6.9/7 (SD 0.4) for ease of learning, and 6.5/7 (SD 0.3) for satisfaction	
Krok-Schoen, 2019 [11]	97.3% of patients reported a positive experience (out of *n =* 37)	12/39 did not complete the intervention for the following reasons: being busy, not feeling well, or forgetfulness
Maguire, 2015 [37]	All patients agreed the handset helped them manage symptoms and communicate with the physician/nurses	100% reported that they encountered problems in using the handset
Mougalian, 2017 [38]	73% of respondents reported that the text messages helped them take their medication either very much or quite a lot.	4.7% of respondents felt the intervention took up too much time
Rico, 2017 [39]	*n =* 15 reported being satisfied or very satisfied	
Wells, 2020 [42]	Of the 13 patients who used smart messaging and were interviewed found smart messages to be a prompt and reminder, some also found it motivating or drew patients back to mindfulness, second theme of personal connection was found (i.e., “someone is thinking about me”) even when patient knew the message wasn’t personally sent	Two patients explained opting out due to lack of confidence in mobile phones

^a^ Patient satisfaction outcomes: The following hierarchy was used to determine which patient satisfaction outcomes to include in the table: (1) overall satisfaction, (2) text message-specific satisfaction. ^b^ Barrier outcomes: The following hierarchy was used to determine which barriers outcomes to include in the table: (1) barriers related to the intervention, (2) barriers related to recruitment. Abbreviations: usability, satisfaction, and ease of use (USE). Note: outcome sample sizes are equal to sample sizes at baseline unless otherwise specified. For all outcomes, when multiple time points were recorded, we reported the longest available follow-up.

**Table 3:** Symptom (*n =* 6 studies) and quality of life (*n =* 10 studies) outcomes.

Author, Year	Symptom Outcomes ^a^	QoL Outcomes ^b^
Randomized Trials		
Gomersall, 2019 [27]		Mean change score (Week 12-Week 4) for time prolonged sitting (min/16 h awake) in I vs. C: −24.4, 95% CI −47.7, −1.1 (within-group *p =* 0.04) vs. 0.0, 95% CI −24.8, 24.7 (within-group *p =* 1)
Haggerty, 2017 [28]		Median 6-month change score for SF-12 physical health component in I vs. C1 vs. C2: 0.9 IQR −0.7–4.8 (*n =* 11) vs. 5.4 IQR 3.8–15.0 (*n =* 11) vs. 7.4 IQR 1.8–11.0 (*n =* 10); *p =* 0.04 between I and C1; Median 6-month change score for Multidimensional Body Self Relations Questionnaire-Appearance subscale in I vs. C1 vs. C2: 0.0 IQR −1.0, 0.0 (*n =* 11) vs. −3.5 IQR −5.0, −1.0 (*n =* 11) vs. −0.5 IQR 1.5, 0 (*n =* 10); *p =* 0.035 between I and C1
Rico, 2020 [30]	Number of patients experiencing side effects experienced in cycle 3, I vs. C: 0–3 side effects 19 vs. 15, 4–14 side effects 24 vs. 29, *p =* 0.38	
Spoelstra, 2016 [34]	Mean total number of symptoms in I vs. C: 4.9 SD 0.4 vs. 5.2 SD 0.6, *p =* 0.7 (ES 0.09)	Mean PROMIS Physical function score in I vs. C: 45.7 SD 0.9 vs. 45.7 SD 1.3, *p =* 0.99 (ES 0); Mean PROMIS Depression score in I vs. C: 44.6 SD 1.0 vs. 44.2 SD 1.3, *p =* 0.8 (ES 0.06)
Spoelstra, 2015 [33]	Mean total number of symptoms in I vs. C: 3.9 SD 0.5 vs. 5.3 SD 0.5, *p =* 0.04 (ES 0.5)	Mean PROMIS Physical function score in I vs. C: 47.6 SD 1.2 vs. 44.9 SD 1.1, *p =* 0.1 (ES 0.4); Mean PROMIS Depression score in I vs. C: 44.7 SD 1.3 vs. 44.9 SD 1.2, *p =* 0.9 (ES 0.03)
Villaron, 2018 [32]		Mean QLQ-30 Physical capacity score at Week 8 in I vs. C: 88.2 SD 13.6 vs. 83.6 SD 12.7, *p =* 0.3; Mean MFI-20 Mental fatigue score at Week 8 in I vs. C: 6.9 SD 3.8 vs. 10.0 SD 4.2, *p* < 0.05
Non-Randomized Interventional/Observational Studies		
Bade, 2018 [35]		Mean daily step count Week 0 vs. Week 3: I group (*n =* 15) 4906.1 SD 256.8 vs. 5241.2 SD 291.7 (ES 0.02); C group (*n =* 22) 5128.2 SD 223.7 vs. 5247.2 SD 242.9 (ES 0.05)
Chow, 2019 [36]		Mean PHQ-4 score was 1.7 SD 2.3 (*n =* 9 reported at least a moderate level of distress ≥ 6 at any point)
Krok-Schoen, 2019 [11]	Mean Breast Cancer Prevention Trial Symptom Checklist score not significantly different between I vs. C (*n =* 37): 0.8 SD 0.5 vs. 0.7 SD 0.5, MD (I–C) 0.04, 95% CI −0.06, 0.1, *p =* 0.4	Mean SF-8 physical health component score I vs. C (*n =* 36): 46.4 SD 10.6 vs. 45.4 SD 10.3, MD (I–C) 1.0, 95% CI −1.7, 3.6, *p =* 0.4; Mean SF-8 mental health component score I vs. C (*n =* 36): 53.0 SD 6.5 vs. 49.9 SD 7.8, MD (I–C) 3.0, 95% CI 0.9, 5.1, *p =* 0.007
Maguire, 2015 [37]	Median ESAS nausea score in I vs. C (*n =* 16): 2, range 0–6 vs. 0, range 0–8	Median ESAS depression score in I vs. C (*n =* 16): 0, range 0–8 vs. 0, range 0–8
Mougalian, 2017 [38]	Number of patients reporting any symptoms for Tamoxifen patients vs. AI patients vs. all patients: 35 vs. 56 vs. 91 (*p =* 0.20 for Tamoxifen vs. AI patients)	
Wells, 2020 [42]		Depression (PHQ-9) reduced by 2.3 points (95% CI: 0.76–3.89) *p =* 0.004

^a^ Symptom outcomes: The following hierarchy was used to determine which symptom outcome to include in the table: (1) composite symptom scores, (2) nausea if acute population or pain if follow-up population (3) fever if acute population or shortness of breath if follow-up population, (4) other relevant outcomes. ^b^ QoL outcomes: The following hierarchy was used to determine which QoL outcomes to include in the table: (1) QoL outcome if present, (2) one physical health outcome (functional status, physical activity, diet) and one mental health outcome (mood, depression anxiety). Abbreviations: aromatase inhibitor (AI), confidence interval (CI), control group (C), effect size (ES), Edmonton Symptom Assessment System (ESAS), intervention group (I), interquartile range (IQR), mean difference (MD), Multidimensional Fatigue Inventory (MFI), Patient Health Questionnaire (PHQ), Patient-Reported Outcomes Measurement Information System (PROMIS), quality of life (QoL), Quality of Life Questionnaire (QLQ), standard deviation (SD), short form (SF). Note: outcome sample sizes are equal to sample sizes at baseline unless otherwise specified. For all outcomes, when multiple time points were recorded, we reported the longest available follow-up.

**Table 4:** Feasibility and implementation outcomes (*n* = 16 studies).

Author, Year	Feasibility Outcomes ^a^
Randomized Trials	
Casillas, 2019 [26]	Mean survivorship care knowledge scale total score (range 1–5) in I vs. C1 vs. C2: 3.8 SD 0.9 pre, 4.2 SD 0.8 post (within-group *p* < 0.05) vs. 4.0 SD 0.9 pre, 4.1 SD 0.8 post (within-group *p* = 0.38); 3.5 SD 0.8 pre, 3.4 SD 0.6 post (within-group *p* = 0.67), *p* < 0.05 for I vs. C2 (ES 0.7), *p* = 0.07 (ES 0.3) for C1 vs. C2
Gomersall, 2019 [27]	83% (*n* = 31) of patients attended all four exercise sessions
Hershman, 2020 [29]	Medication adherence failure (based on urine samples, accounting for censoring) in I vs. C: total 283 vs. 303 events, HR 0.9, 95% CI 0.8, 1.1, *p* = 0.2
Rico, 2020 [30]	52 text messages were sent from day 1 to the beginning of cycle 4
Spoelstra, 2016 [34]	Mean adherence to OA in I vs. C: 6.5 SD 0.4 vs. 7.2 SD 0.5, *p* = 0.3 (ES 0.3)
Spoelstra, 2015 [33]	Overall mean adherence in I vs. C: 6.0 SD 0.5 vs. 6.0 SD 0.5, *p* = 1 (ES 0); 1359 texts were sent to patients (1111 adherence, 116 symptom management, 52 additional, 53 welcome and 17 end of study)
Tan, 2020 [31]	SMAQ adherence in I vs. C vs. All: 52.0% vs. 54.6% vs. 53.3%
Villaron, 2018 [32]	Survey compliance was 64.6%
Non-Randomized Interventional/Observational Studies	
Bade, 2018 [35]	Number of patients never using the device in I vs. C: 0% vs. 21% (out of *n* = 15 vs. *n* = 29)
Chow, 2019 [36]	Screener adherence rate was 75%
Krok-Schoen, 2019 [11]	Mean Morisky Adherence score in I vs. C (*n* = 36): 1.2 SD 1.3 vs. 1.9 SD 1.7, MD (I–C) −0.8, 95% CI −1.4, −0.2, *p* = 0.02
Maguire, 2015 [37]	182 alerts were generated over 12 months (138 amber, 44 red)
Mougalian, 2017 [38]	86.1% of patients responded to all the daily texts (among those who completed the pilot study, response rate was 92.2%); Average of 10 min/week was spent using the application
Sawicki, 2019 [40]	40% response rate to texts requiring a response; Optimal adherence in I vs. C: 53.4% vs. 43.7% (difference 9.7%, *p* = 0.02)
Tan, 2019 [41]	No show rate in C vs. I: adjusted OR 6.8, 95% CI 5.5, 8.4, *p* < 0.0001
Wells, 2020 [42]	Odds of completing MBCT in I vs. C: 87% vs. 38%, *p* = 0.007, adjusted OR 7.8, 95% CI 1.8, 34.6

^a^ Feasibility outcomes: The following hierarchy was used to determine which feasibility outcomes to include in the table: (1) one patient-level outcome and one systems-level outcome if available. Abbreviations: confidence interval (CI), control group (C), effect size (ES), intervention group (I), mean difference (MD), mindfulness-based cognitive therapy (MBCT), odds ratio (OR), oral anticancer (OA), standard deviation (SD), Simplified Medication Adherence Questionnaire (SMAQ). Note: outcome sample sizes are equal to sample sizes at baseline unless otherwise specified. For all outcomes, when multiple time points were recorded, we reported the longest available follow-up.

## Data Availability

Not applicable.

## Supplementary Materials

The following are available online at https://www.mdpi.com/article/10.3390/cancers13143542/s1. Figure S1: Bias assessment of the randomized controlled trials (*n* = 9). Figure S2: Bias assessment of the non-randomized interventional/observational studies (*n* = 9). Table S1: Study inclusion and exclusion criteria. Table S2: Search strategy for included databases. Table S3: Detailed characteristics and outcomes of included studies (*n* = 18). Table S4: Cochrane Risk of Bias table for the randomized controlled trials (*n* = 9). A score of 0 represents high risk of bias, 1 represents unclear risk of bias, and 2 represents low risk of bias. Table S5: Risk of Bias in Non-randomized Studies-of Interventions (ROBINS-I) table for the non-randomized interventional/observational studies (*n* = 9).
