# Supplementary material for: Text Messaging in Cancer-Supportive Care: A Systematic Review
Source: Cancers (Basel). 2021 Jul 15;13(14):3542. doi: 10.3390/cancers13143542 (PMC8307703; doi:10.3390/cancers13143542)
Supplement: Supplementary file 1 [file cancers-13-03542-s001.zip › cancers-1237664-supplementary.pdf]

cancers

MDPI

Supplementary Materials: Text Messaging in Cancer Supportive Care: A Systematic Review

Don Thiwanka Wijeratne, Meghan Bowman, Isobel Sharpe, Siddhartha Srivastava, Matthew Jalink and Bishal Gyawali

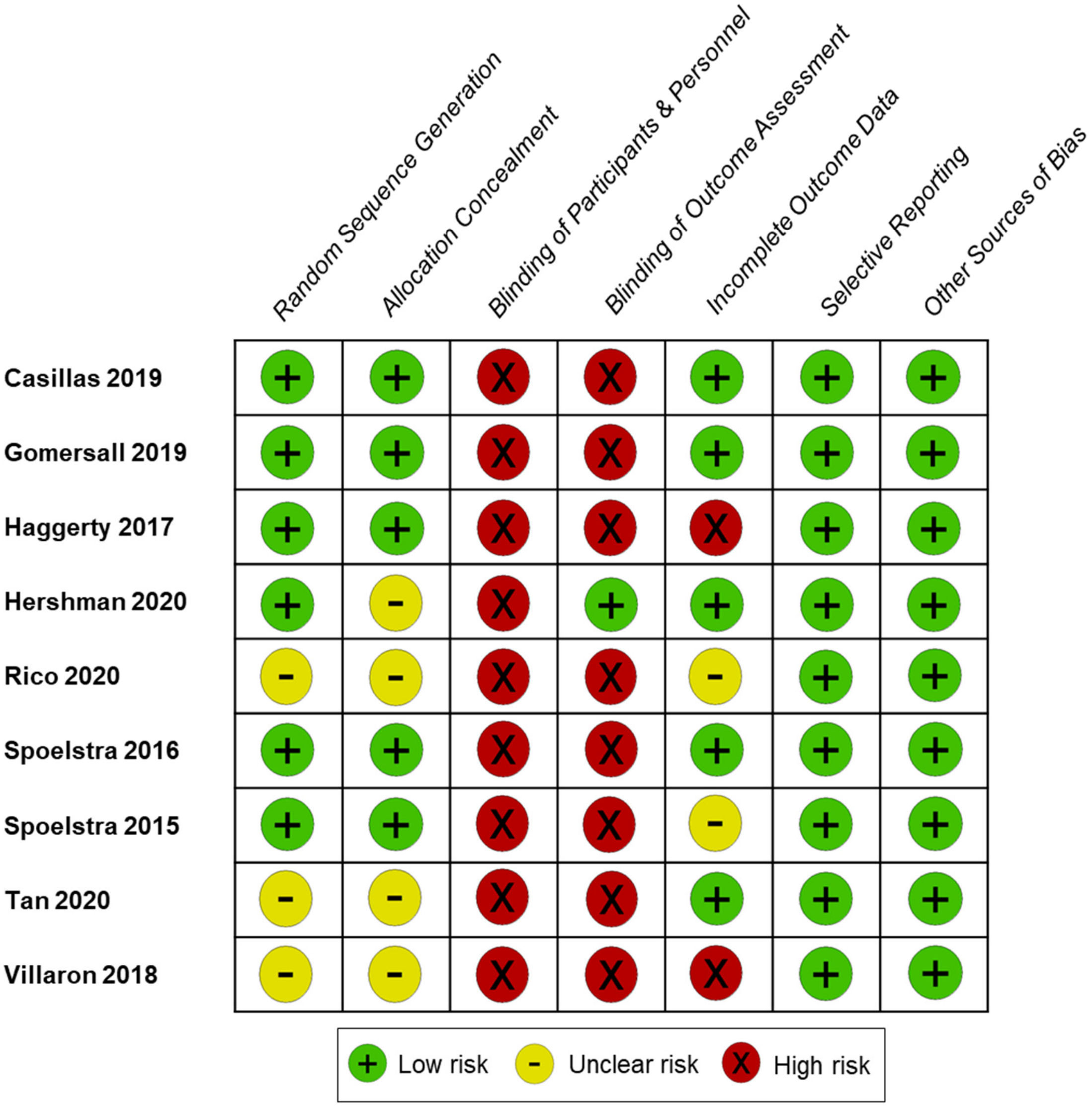

Figure S1. Bias assessment of the randomized controlled trials ($n = 9$).

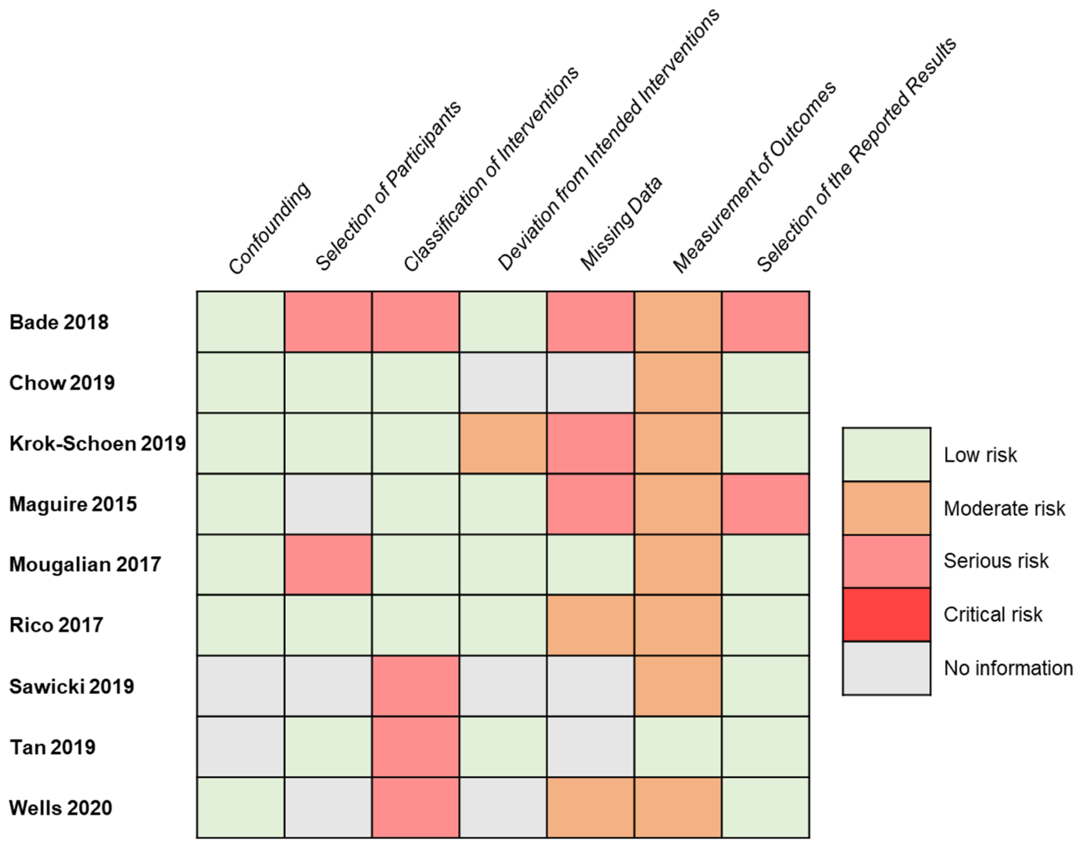

Figure S2. Bias assessment of the non-randomized interventional/observational studies (n = 9).

Table S1. Study inclusion and exclusion criteria.

Criteria	Inclusion	Exclusion
Population	Adult cancer patients (age ≥ 18 years)	No diagnosis of cancer<br>Perioperative cancer patients
Intervention	Main study intervention is the use of text-based communication in cancer supportive care	No use of text-based communication<br>Use of modalities other than text messaging as the main study intervention (e.g., in-person visits, telephone calls)<br>Multi-modal interventions that include text-based communication (cannot discern text-specific effects)<br>Intervention development/ single-use studies
Control	Any	
Outcome	Symptom control<br>Quality of life<br>Survival<br>Resource utilization<br>Cost<br>Feasibility<br>Training<br>Satisfaction<br>Safety	
Study Design	Primary studies involving patients (any design):<br>Clinical trials<br>Observational studies	Commentaries/opinion<br>Case studies<br>Case series<br>Reviews<br>No full text available (abstract only)

Table S2. Search strategy for included databases.

Database	Search Strategy	Results
MEDLINE	S1 exp mHealth/(30756)<br>S2 exp mobile health/(30756)<br>S3 *mobile healthcare/(0)<br>S4 *two-way communication/(0)<br>S5 *text-based communication/(0)<br>S6 *SMS messaging/(0)<br>S7 exp text messaging (3081)<br>S8 1 or 2 or 3 or 4 or 5 or 6 or 7 (33359)<br>S9 exp cancer/(3381158)<br>S10 *cancer supportive care/(0)<br>S11 *cancer care/(0)<br>S12 *supportive care in cancer/(0)<br>S13 exp chemotherapy/(1371859)<br>S14 *side effects/(0)<br>S15 exp adverse effects/(599)<br>S16 *integrated care/(0)<br>S17 *cancer integrated care/(0)<br>S18 exp treatment adherence/(247747)<br>S19 exp neoplasms/(3381490)<br>S20, 9 or 10 or 11 or 12 or 13 or 14 or 15 or 16 or 17 or 18 or 19 (4627996)<br>S21 exp quality of life/(199629)<br>S22 *quality of service/(0)<br>S23 exp quality of care/(7055796)<br>S24 exp healthcare delivery/(1094179)<br>S25 *healthcare management/(0)<br>S26 exp care management/(796912)<br>S27 exp continuity of care/(248867)<br>S28 *lean healthcare/(0)<br>S29 *lean health care/(0)<br>S30 *lean thinking/(0)<br>S31 *patient-centered/(0)<br>S32 *patient self management/(0)<br>S33 exp self management/(2561)<br>S34 21 or 22 or 23 or 24 or 25 or 26 or 27 or 28 or 29 or 30 or 31 or 32 or 33 (7797450)<br>S35 20 and 34 (1750932)<br>S36 20 or 35 (4628439)<br>S37 8 and 36 (6114)<br>S38 limit 35 to (english language and humans and yr = "2015 - Current") (3207)	3207
CINAHL	S1 (MH "Text Messaging+") (3081)<br>S2 "mhealth" (15762)	459

	S3 "mobile healthcare" (98)<br>S4 "two-way communication" (135)<br>S5 "text-based communication" (24)<br>S6 "SMS messaging" (35)<br>S7 (MH "Neoplasms+")(555537)<br>S8 "cancer" (430389)<br>S9 "cancer supportive care" (39)<br>S10 "Supportive care in cancer" (237)<br>S11 "side effects" (38892)<br>S12 "adverse effects" (473751)<br>S13 "integrated care" (4488)<br>S14 "cancer integrated care" (2)<br>S15 "treatment adherence" (3003)<br>S16 (MH "Quality of life+") OR (MH "Health care delivery+") OR (MH "Self Management+") (460370)<br>S17 "quality of service" (994)<br>S18 "quality of care" (60427)<br>S19 "healthcare management" (530)<br>S20 "care management" (6036)<br>S21 "continuity of care" (10721)<br>S22 "lean healthcare" (38)<br>S23 "lean health care" (14)<br>S24 "lean thinking" (119)<br>S25 "patient centered care"(32224)<br>S26 "patient self management" (623)<br>S27 S1 OR S2 OR S3 OR S4 OR S5 OR S6 (18716)<br>S28 S7 OR S8 OR S9 OR S10 OR S11 OR S12 OR S13 OR S14 OR S15 (1097631)<br>S29 S16 S17 S18 S19 S20 S21 S22 S23 S24 S25 S26 (538085)<br>S30 S28 AND S29 (62859)<br>S31 S30 OR S28 (1097631)<br>S32 S31 AND S27 limit to 20150101-20201231; English Language; Human (459)	
EMBASE	S1 *mHealth/(21)<br>S2 *mobile health/(96)<br>S3 *mobile healthcare/(7)<br>S4 *two-way communication/(0)<br>S5 *text-based communication/(0)<br>S6 *SMS messaging/(0)<br>S7 exp text messaging/ (5334)<br>S8 1 or 2 or 3 or 4 or 5 or 6 or 7 (5426)<br>S9 exp cancer/(3781573)<br>S10 *cancer supportive care/(6)<br>S11 *cancer care/(84)<br>S12 *supportive care in cancer/(1)<br>S13 exp chemotherapy/(691694)	783

S14 *side effects/(0)
S15 exp adverse effects/(654856)
S16 *integrated care/(27)
S17 *cancer integrated care/(0)
S18 exp treatment adherence/(161688)
S19 exp neoplasms (5016276)
S20 9 or 10 or 11 or 12 or 13 or 14 or 15 or 16 or 17 or 18 or S19 (5774245)
S21 exp quality of life/(509286)
S22 *quality of service/(6)
S23 exp quality of care/(3333042)
S24 exp healthcare delivery/(3480453)
S25 exp healthcare management/(1346524)
S26 *care management/(2)
S27 exp continuity of care/(849670)
S28 *lean healthcare/(2)
S29 *lean health care/(0)
S30 *lean thinking/(8)
S31 *patient-centered/(66189)
S32 *patient self management/(6)
S33 exp self management/(85482)
S34 21 or 21 or 22 or 23 or 24 or 25 or 26 or 27 or 28 or 29 or 30 or 31 or 32 or 33 (6922240)
S35 20 and 34 (1544870)
S36 20 or 35 (4586432)
S37 8 and 36 (1053)
S38 limit 37 to (english language and humans and yr = "2015 - Current") (783)

Table S3. Detailed characteristics and outcomes of included studies (*n* = 18).

Author, Year (Country)	Study Design	Study Population	Baseline Sample Size I vs. C (age mean SD, % male)	Type of Care Provided	Treatment Focus	Intervention Description	Control Description	Follow-up Length
Randomized Trials								
Casillas, 2019 (United States) [26]	RCT (three-armed, parallel, prospective, single-center)	Adolescent and young adult childhood cancer survivors (off cancer treatment for more than one year)	Text messages (I) vs. C1 vs. C2: 28 vs. 25 vs. 25 (I: 21 SD 5, 54%; C1: 21 SD 6, 44%; C2: 20 SD 5, 44%)	Supportive (motivation, self-management education)	Follow-up (survivorship)	Sent 3 goals to the research team by text, which initiated an individualized two-way automated text messaging system. The system was intended to support survivor engagement in accessing community and cancer center resources to help patients reach their goals.<br><br>Note: all groups received an educational booklet and identified 3 survivorship action plan goals.	Peer navigation - C1: received telephone calls at Weeks 0 and 4 to discuss goals<br><br>Control group - C2: encouraged to seek answers to questions regarding the educational material	2 months
Gomersall, 2019 (Australia) [27]	RCT (two-armed, parallel, prospective, single-center; ITT analysis)	Confirmed cancer diagnosis and at least one month post-surgery	18 vs. 18 (I: 68 SD 9, 67%; C: 61 SD 9, 61%)	Supportive (PA motivation)	Follow-up	Completed the same exercise rehabilitation program as controls (4 weeks), and received tailored text messages designed to improve whole-of-day activity (12 weeks). Messages were generated and sent by research staff (minimum 6/fortnight,	Exercise rehabilitation program only (1 hour/ week for 4 weeks)	3 months

						with 2 educational tips, 3 real-time prompts, and 1 goal-check; patients were prompted to reply to the goal-check and responses were checked by research staff). Messages were tailored based on data collected at sessions before and after the exercise program.		
Haggerty, 2017 (United States) [28]	RCT (three-armed, parallel, prospective, multi-center)	Women with history of endometrial cancer (not active), BMI 30+, no current or planned treatments	Text messages (I) vs. C1 vs. C2: 13 vs. 14 vs. 15 (overall: 60 SD 9, 0%)	Supportive (weight loss)	Follow-up	Received 3–5 daily personalized interactive text messages (provided feedback, support, strategies for long-term behaviour change, and a once per week prompt to record weight using a conventional scale).<br><br>Note: both the text message group and TM group were encouraged to meet the same calorie and exercise goals and to record all food and beverage intake.	TM group - C1: received weekly/ bi-weekly telephone counselling, recorded weight using a WiFi-enabled scale.<br><br>Control group - C2: received paper handouts on healthy eating and exercise.	6 months
Hershman, 2020 (United States) [29]	RCT (two-armed, parallel, prospective, multi-center; ITT analysis)	Post-menopausal women with breast cancer (stage I–	348 vs. 354 (I: median 61 SD 7, 0%; C: median 60 SD 8, 0%)	Supportive (medication adherence)	Hormone therapy	Received twice-weekly educational text messages (one on a weekday and one on a weekend) focusing on overcoming potential barriers to medication adherence: included cues	No text messaging	3 years

		III) taking a third generation aromatase inhibitor				to action, statements related to the efficacy of the medication, reinforcements of physician recommendations, and words of support and encouragement. Messages were randomly selected out of a predetermined set of 40 (developed based on literature review and focus groups).		
Rico, 2020 (Brazil) [30]	RCT (two-armed, parallel, prospective, single-center)	Chemotherapy outpatients	59 vs. 59 (I: 37% in 51–60 age group, 51%; C: 37% in 51–60 age group, 37%)	Supportive (self-care and emotional support)	Chemotherapy	Text messages provided content on prevention of side effects and emotional support. Messages were sent automatically on a daily basis using the cHEmotHErAPP. Messages were divided into themes; for each theme various pieces of advice were drafted and sent. Text messaging was timed with treatment period of patients (no messages with same theme were sent for at least 5 consecutive days or repeat messages for at least 45 days.	Standard care with periodic questionnaires	10 months
Spoelstra, 2016 (United States) [34]	RCT (two-armed, paral-	Cancer patients newly	49 vs. 26 (I: 60 SD 10, 47%; C: 60 SD 11., 42%)	Supportive (medication adherence)	OA treatment	Texts were developed and sent according to social cognitive theory. Included	Usual care	9 weeks

	lel, prospective, multi-center)	prescribed OA medication				one welcome text, 6 following daily medication adherence texts used on rotating basis, and one end of study text. Automated system sent messages and stored data.		
Spoelstra, 2015 (United States) [33]	RCT (two-armed, parallel, prospective, multi-center)	Cancer patients newly prescribed OA medication	40 vs. 40 (I: 59 SD 11, 43%; C: 58 SD 10, 38%)	Supportive (medication adherence)	OA treatment	Texts were developed and sent according to social cognitive theory. Included one welcome text, 6 following daily medication adherence texts used on rotating basis, weekly symptom management texts, and one end of study text. Automated system sent messages and stored data. At the end of the intervention patients were asked if they wanted to continue for another week.	Usual care	9 weeks
Tan, 2020 (Singapore) [31]	RCT (two-armed, parallel, prospective, multi-center)	Women with breast cancer, 21+ years old, prescribed AET for at least 1 year	123 vs.121 (I: median 60 range 32–80, 0%; C: median 62 range 39–80, 0%)	Supportive (medication adherence)	Hormone therapy	SMS reminders to take anti-cancer medication were sent weekly on Mondays at 9 am in English, Mandarin, or Malay.	Routine clinical follow-up without text-based reminders	1 year

Villaron, 2018 (France) [32]	RCT (two-armed, parallel, prospective, single-center)	Chemotherapy outpatients capable of practice-adapted physical activity (walking)	21 vs. 22 (I: 48 SD 11, 10%; C: 51 SD 15, 46%)	Supportive (PA motivation)	Chemotherapy	Patients received advice on increasing their level of physical activity via recommendation guide and motivational text messages sent at the beginning of every week to encourage physical activity. These underlined the need to read and refer to the guide booklet for recommendations.<br><br>Note: both groups wore a pedometer every day and filled out an online questionnaire.	No recommendation booklet or text messages	2 months
Non-Randomized Interventional/Observational Studies								
Bade, 2018 (United States) [35]	Comparative	Advanced-stage lung cancer (III or IV)	15 vs. 29 (I: 64 SD 9, 60%; C: 68 SD 8, 77%)	Supportive (PA motivation)	Any (diagnosis, during, or after treatment)	Received twice-daily personalized text messages. Messages included: weekly activity goal, current step count, and motivational statements (12 weeks total). Also participated in a 20-minute exercise education session upon enrolment.<br><br>Note: both groups wore FitBits to track activity goals.	Weekly phone calls (delivered activity goals, reminders to exercise; 4 weeks total)	1 month

Chow, 2019 (United States) [36]	Single arm observational	Receiving active cancer treatment	52 (58, 38%)	Supportive (distress)	Chemotherapy	Received text message invitation to complete a web-based distress screener (the PHQ-4) once per week for 4 weeks. In event of a high distress score (>9), an automatic email was sent to the patient's PCP.	None	1 month
Krok-Schoen, 2019 (United States) [11]	Pre-post (pilot)	Post-menopausal women with breast cancer (stage 0–III) eligible to receive adjuvant hormone therapy for the first time	39 (60 SD 7, 0%)	Supportive (medication adherence)	Hormone therapy	Received daily text message reminders to take hormone therapy medication (selected from a library of 14 distinct positive adherence messages). Also received a weekly text message prompt to complete a medication adherence survey within a mobile app (asking "over the past 7 days, how many days did you take your medication?"). The treating physician was notified by email of patients who missed more than one dose.	Study sample at baseline	3 months
Maguire, 2015 (United Kingdom) [37]	Single-arm mixed methods	Lung cancer patients receiving thoracic radiotherapy	16 (64 SD 13, 25%)	Symptom monitoring, supportive (self-care advice)	Radiation therapy	ASyMS (mobile phone-based system used for remote patient monitoring): daily symptom questionnaires were completed data were then sent in real time to a central study	None	Length of radiotherapy + 1 additional month

						server and an integrated risk model analyzed and reported symptoms. Patients immediately received self-care advice on their mobile phone (directly applied to the severity of their symptoms), and the server also generated alerts to a pager held by a health professional at the clinic.		
Mougalian, 2017 (United States) [38]	Pilot	HR-positive breast cancer patients (stage I to III), recommended adjuvant hormonal therapy	100 vs.100 (I: median 54 IQR 47–62, 0%; C: median 55 IQR 46–65, 0%)	Symptom monitoring, supportive (medication adherence, prescription reminders)	Follow-up	BETA-Text: generated texts via CarePlanManager (developed by CircleLinkHealth). Consisted of 3 types of text messages to which patients responded: daily medication reminders, weekly AE questions, and monthly texts regarding barriers to adherence. Generated alerts were forwarded to the clinical team if the patient reported 3 consecutive missed text responses (considered missed doses), more than 6 missed within the previous 30 days, or severe AEs (7–9 on the severity scale). Participants also received	Standard care: historical controls using medical records	3 months

						reminder texts to refill prescriptions.		
Rico, 2017 (Brazil) [39]	Pilot	Chemotherapy outpatients	14 (44 range 21–68, 36%)	Supportive (self-care and emotional support)	Chemotherapy	cHEmotHErApp automatically sent patients daily text messages with guidelines that promoted self-care and emotional support. The app allowed registration of patient data and chemotherapy schedules and orientations, and included a 4 table data model. All messages were stored in SMS app on the patient's smartphone. In case a message was not received by a patient an error message was shown.	None	1 month
Sawicki, 2019 (United States) [40]	Retrospective cohort	Patients initiated on TKI therapy (imatinib mesylate, dasatinib, bosutinib, or nilotinib)	279 vs. 279 (I: 53 SD 13, 52%; C: 54 SD 15, 80%)	Supportive (prescription reminders, appointment booking reminders, coaching, status updates)	Follow-up	A number of message categories were included such as lab testing, adherence to prescribed therapy, symptoms and side effects, and condition-specific management guidance. Pertinent messages were shared with the patients at the points in time when they were most appropriate. Messages included a hyperlink so patients could request additional consultations with a	Patients who could not be reached via phone by patient service representatives were enrolled in 1-way texting	1 year

						specifically trained pharmacist. Some messages prompted patient responses and were sent at 3,6,9,12 months in accordance with recommended safety measures.		
Tan, 2019 (United States) [41]	Retrospective analysis	Cancer patients undergoing radiation therapy	668 vs. 2761 (I: 65% in 30–64 age group, 44%; C: 58% in 30–64 age group, 53%)	Supportive (appointment reminders)	Radiation therapy	Custom SMS platform connected to medical records that automatically sent SMS messages concerning radiation therapy appointments. Daily text reminders sent 2 hours prior to their appointment with appointment-specific information.	None	7 months
Wells, 2020 (United Kingdom) [42]	Mixed methods proof of concept	Cancer patients from hospital/ palliative care psycho-oncology service, currently receiving treatment, experiencing mild to moderate clini-	30 vs. 21 (I: 54 SD 8, 17%; C: 59 SD 13, 14%)	Supportive (session and practice reminders, education)	Active treatment	Patients who opted in received MBCT intervention (focused on mindfulness skills, specifically the use of non-judgmental, present moment awareness to make purposeful choices re: self-management of physical and emotional health). Sessions also reviewed activities done between sessions. Text messages were sent a day after the MBCT session reminding of home practice. Two days later, a reminder on the theme of	MBCT sessions, opted out of smart messaging	1 month

cal anxiety and/or depressive symptoms	the previous session was sent. A reminder was also sent the day before each session.

Table S3. (cont'd): Detailed characteristics and outcomes of included studies (*n* = 18).

Author, Year	Symptom Control	Quality of Life (inc. diet, exercise, mental health, weight)	Feasibility
Randomized Trials			
Casillas, 2019 [26]			Note: all outcomes in I vs. C1 vs. C2 were *n* = 23 vs. *n* = 24 vs. *n* = 24.<br><br>Know the term "late effects" I vs. C1 vs. C2: 74% pre, 100% post (within-group $p < 0.05$) vs. 83% pre, 92% post (within-group $p = 0.50$) vs. 83% pre, 92% post (within-group $p = 0.69$), $p = 1$ for I vs. C2, $p = 1$ for C1 vs. C2.<br><br>Mean scores on survivorship care knowledge scale (truthfulness of statements range 1–5) I vs. C1 vs. C2: the reason for survivorship care is to check for recurrence 4.2 SD 1.2 pre, 4.5 SD 0.8 post (within-group p = 0.1) vs. 4.5 SD 0.9 pre, 4.7 SD 0.8 post (within-group p = 0.20) vs. 4.3 SD 1.1 pre, 4.1 SD 1.1 post (within-group p = 0.4), $p < 0.05$ (ES 0.5) for I vs. C2, $p = 0.1$ (ES 0.4) for C1 vs. C2; the reason for survivorship care is to obtain advice on how cancer treatment may affect health 4.4 SD 0.9 pre, 4.7 SD 0.6 post (within-group $p = 0.1$) vs. 4.4 SD 0.9 pre, 4.5 SD 0.8 post (within-group $p = 0.4$) vs. 3.9 SD 0.9 pre, 4.0 SD 0.9 post (within-group $p = 0.5$), $p = 0.05$ (ES 0.6) for I vs. C2, $p = 0.4$ (ES 0.3) for C1 vs. C2; the reason for survivorship care is to obtain emotional/psychological support 2.8 SD 1.7 pre, 3.5 SD 1.4 post (within-group $p = 0.09$) vs. 3.0 SD 1.7 pre, 3.0 SD 1.8 pos (within-group $p = 0.9$) vs. 2.3 SD 1.3 pre, 2.2 SD 1.0 post (within-group $p = 0.7$), $p < 0.05$ (ES 0.8) for I vs. C2, p = 0.3 (ES 0.3) for C1 vs. C2; scale total score 3.8 SD 0.9 pre, 4.2 SD 0.8 post (within-group $p < 0.05$) vs. 4.0 SD

	0.9 pre, 4.1 SD 0.8 post (within-group $p = 0.4$); 3.5 SD 0.8 pre, 3.4 SD 0.6 post (within-group $p = 0.7$), $p < 0.05$ (ES 0.7) for I vs. C2, $p = 0.07$ (ES 0.3) for C1 vs. C2<br><br>Mean scores on survivorship care attitudes scale (importance of statements range 1–5) I vs. C1 vs. C2: having copy of survivorship care plan 4.3 SD 1.0 pre, 4.4 SD 1.0 post (within-group $p = 0.6$) vs. 4.4 SD 0.8 pre, 4.8 SD 0.4 post (within-group $p < 0.05$) vs. 4.0 SD 1.3 pre, 4.0 SD 1.2 post (within-group $p = 1$), $p = 0.3$ (ES 0.3) for I vs. C2, $p < 0.05$ (ES 0.7) for C1 vs. C2; having medical care related to cancer treatment and late effects 4.6 SD 0.7 pre, 4.8 SD 0.5 post (within-group $p < 0.05$) vs. 4.7 SD 0.7 pre, 4.8 SD 0.6 post (within-group $p = 0.3$) vs. 4.5 SD 0.7 pre, 4.2 SD 0.9 post (within-group $p = 0.05$), $p < 0.05$ (ES 0.7) for I vs. C2, $p < 0.05$ (ES 0.5) for C1 vs. C2; taking better care of health compared to peers never treated for cancer 4.5 SD 0.8 pre, 4.7 SD 0.6 post (within-group $p = 0.2$) vs. 4.4 SD 0.8 pre, 4.7 SD 0.6 post (within-group $p = 0.1$) vs. 4.0 SD 1.0 pre, 4.0 SD 0.9 post (within-group $p = 1$), $p < 0.05$ (ES 0.2) for I vs. C2, $p = 0.06$ (ES 0.2) for C1 vs. C2; having health insurance coverage as a cancer survivor 4.8 SD 0.6 pre, 5.0 SD 0.2 post vs. 4.8 SD 0.4 pre, 5.0 SD 0.0 post (within-group $p < 0.05$) vs. 4.8 SD 0.5 pre, 4.7 SD 0.7 post (within-group $p = 0.5$), $p = 0.06$ (ES 0.6) for I vs. C2, $p = 0.06$ (ES 0.6) for C1 vs. C2; scale total score 4.5 SD 0.7 pre, 4.7 SD 0.4 post (within-group $p = 0.07$) vs. 4.6 SD 0.5 pre, 4.8 SD 0.3 post (within-group $p < 0.05$) vs. 4.3 SD 0.7 pre, 4.2 SD 0.7 post (within-group $p = 0.4$), $p < 0.05$ (ES 0.3) for I vs. C2, $p < 0.05$ (ES 0.4) for C1 vs. C2<br><br>Mean scores on late-effects self-efficacy scale (confidence in statements range 1–5) I vs. C1 vs. C2: how long to continue screening for recurrence 3.8 SD 1.2

pre, 3.7 SD 1.2 post (within-group $p = 0.5$) vs.3.6 SD 1.2 pre, 4.2 SD 0.9 post (within-group $p < 0.05$) vs. 3.5 SD 1.2 pre, 3.6 SD 1.0 post (within-group $p = 0.8$), $p = 0.7$ (ES -0.08) for I vs. C2, $p = 0.05$ (ES 0.4) for C1 vs. C2; steps to take if concerned about physical late effects 3.7 SD 1.2 pre, 3.6 SD 1.1 post (within-group $p = 0.9$) vs. 3.2 SD 1.2 pre, 4.0 SD 1.1 post (within-group $p < 0.05$) vs. 3.3 SD 1.3 pre, 3.3 SD 0.9 post (within-group $p = 1$), $p = 0.5$ (ES 0.2) for I vs. C2, $p < 0.05$ (ES 0.7) for C1 vs. C2; steps to take if concerned about psychological/emotional/social late effects 3.6 SD 1.0 pre, 2.6 SD 1.1 post (within-group $p = 1$) vs. 3.1 SD 1.2 pre, 3.8 SD 1.1 post (within-group $p < 0.05$) vs. 3.1 SD 1.2 pre, 3.3 SD 0.8 post (within-group $p = 0.6$), $p = 0.6$ (ES 0.1) for I vs. C2, $p < 0.05$ (ES 0.6) for C1 vs. C2; scale total score 3.7 SD 1.0 pre, 3.6 SD 1.0 post (within-group $p = 0.7$) vs. 3.3 SD 1.1 pre, 4.0 SD 1.0 post (within-group $p<0.05$) vs. 3.3 SD 1.1 pre, 3.4 SD 0.8 post (within-group $p = 0.8$), $p = 0.8$ (ES 0.05) for I vs. C2, $p < 0.05$ (ES 0.7) for C1 vs. C2

Mean scores on survivorship care planning self-efficacy scale (confidence in statements range 1–5) I vs. C1 vs. C2: obtain own copy of medical records 4.2 SD 1.1 pre, 4.1 SD 1.0 post (within-group $p = 0.8$) vs. 4.1 SD 1.1 pre, 4.4 SD 1.1 post (within-group $p = 0.2$) vs. 3.9 SD 1.2 pre, 3.7 SD 1.1 post (within-group $p = 0.4$), $p = 0.3$ (ES 0.3) for I vs. C2, $p < 0.05$ (ES 0.6) for C1 vs. C2; obtain own copy of treatment summary 4.0 SD 1.2 pre, 4.2 SD 0.9 post (within-group $p = 0.6$) vs. 4.1 SD 1.1 pre, 4.5 SD 0.9 post (within-group $p = 0.2$) vs. 3.9 SD 1.2 pre, 3.8 SD 1.1 post (within-group $p = 0.5$), $p = 0.2$ (ES 0.4) for I vs. C2, $p < 0.05$ (ES 0.7) for C1 vs. C2; obtain own copy of survivorship care plan 4.0 SD 1.1 pre, 4.3 SD 1.1 post (within-group $p = 0.4$) vs. 3.9 SD 1.2 pre, 4.3 SD 1.3 post (within-group $p = 0.2$) 3.9 SD 1.2 pre, 3.7 SD 1.0 post

(within-group $p = 0.3$), $p = 0.05$ (ES 0.5) for I vs. C2, $p < 0.05$ (ES 0.7) for C1 vs. C2; scale total score 4.0 SD 1.1 pre, 4.2 SD 0.9 post (within-group $p = 0.6$) vs. 4.0 SD 1.1 pre, 4.4 SD 1.0 post (within-group $p = 0.1$) vs. 3.9 SD 1.2 pre, 3.7 SD 1.0 post (within-group $p = 0.3$), $p = 0.1$ (ES 0.4) for I vs. C2, $p < 0.05$ (ES 0.7) for C1 vs. C2

Mean scores on health insurance self-efficacy scale (confidence in statements range 1–5): I vs. C1 vs. C2: talk to insurance company about current coverage 2.5 SD 1.3 pre, 3.0 SD 1.4 post (within-group $p < 0.05$) vs. 3.5 SD 1.3 pre, 3.8 SD 1.4 post (within-group $p = 0.4$) vs. 3.0 SD 1.1 pre, 3.0 SD 1.0 post (within-group $p = 0.7$), $p = 0.3$ (ES 0.2) for I vs. C2, $p = 0.1$ (ES 0.4) for C1 vs. C2; obtain a copy of health insurance plan 3.0 SD 1.5 pre, 3.4 SD 1.3 post (within-group $p = 0.3$) vs. 3.7 SD 1.2 pre, 3.9 SD 1.2 post (within-group $p = 0.3$) vs. 3.4 SD 1.1 pre, 3.3 SD 1.0 post (within-group $p = 0.8$), ES = 0.2 and $p = 0.5$ for I vs. C2, ES = 0.4 and $p = 0.2$ for C1 vs. C2; find out types of insurance plans accepted by oncologist 3.1 SD 1.4 pre, 3.6 SD 1.3 post (within-group $p = 0.07$) vs. 3.7 SD 1.1 pre, 4.1 SD 1.3 post (within-group $p = 0.1$) vs. 3.3 SD 1.2 pre, 3.4 SD 1.1 post (within-group $p = 0.5$), $p = 0.4$ (ES 0.2) for I vs. C2, $p = 0.2$ (ES 0.4) for C1 vs. C2; discuss insurance options with healthcare team 3.1 SD 1.4 pre, 3.6 SD 1.4 post (within-group $p = 0.07$) vs. 3.6 SD 1.2 pre, 4.3 SD 1.0 post (within-group $p < 0.05$) vs. 3.3 SD 1.1 pre, 3.4 SD 1.2 post (within-group $p = 0.9$), $p = 0.3$ (ES 0.3) for I vs. C2, $p < 0.05$ (ES 0.6) for C1 vs. C2; talk to billing department about medical bills 2.7 SD 1.2 pre, 3.2 SD 1.3 post (within-group $p < 0.05$) vs. 3.4 SD 1.3 pre, 4.0 SD 1.1 post (within-group $p < 0.05$) vs. 3.1 SD 1.1 pre, 3.1 SD 1.2 post (within-group $p = 1$), $p = 0.21$ (ES 0.30) for I vs. C2, $p < 0.05$ (ES 0.7) for C1 vs. C2; scale total score 2.9 SD 1.2 pre, 3.3 SD 1.3 post (within-

			group $p = 0.07$) vs. 3.5 SD 1.0 pre, 4.0 SD 1.1 post (within-group $p < 0.05$) vs. 3.2 SD 1.0 pre, 3.3 SD 1.1 post (within-group $p = 0.8$), $p = 0.2$ (ES 0.3) for I vs. C2, $p < 0.05$ (ES 0.5) for C1 vs. C2
Gomersall, 2019 [27]		PA OUTCOMES:<br>Sample sizes: I group $n = 18$ at Week 4, $n = 17$ at Week 12; C group $n = 18$ at Week 4, $n = 15$ at Week 12<br>Mean time sitting change score Week 12-Week 4 (min/16 h awake) I vs. C: -33.5 95% CI −59.7, −7.3, $p = 0.01$ vs. −2.2, 95% CI −29.9, 25.6, $p = 0.9$, MD (I–C) −31.3, 95% CI −69.5, 6.8, $p = 0.1$; Mean time 20+ min prolonged sitting change score Week 12-Week 4 (min/16 h awake) I vs. C: −24.4, 95% CI −47.7, −1.1, $p = 0.04$ vs. 0.0, 95% CI −24.8, 24.7, $p = 1$, MD (I–C) −24.4, 95% CI −58.4, 9.6, $p = 0.2$; Mean usual sitting bout duration change score Week 12-Week 4 (min) I vs. C: −2.1, 95% CI −4.4,0.2, $p = 0.08$ vs. 1.4, 95% CI −1.0, 3.9, $p = 0.2$, MD (I–C) −3.5, 95% CI −6.9, −0.2, $p = 0.04$; Mean time standing change score Week 12-Week 4 (min/16 h awake) I vs. C: 33.9, 95% CI 2.8,65.0, $p = 0.03$ vs. 6.7, 95% CI −26.4, 39.7, $p = 0.7$, MD (I–C) 27.2, 95% CI −18.1, 72.6, $p = 0.2$; Mean time stepping change score Week 12-Week 4 (min/16 h awake) I vs. C: −1.0, 95% CI −13.8, 11.8, $p = 0.9$ vs. −4.2, 95% CI −17.8,9.3, $p = 0.5$, MD (I–C) 3.3, 95% CI −15.3, 21.9, $p = 0.7$; Mean time light stepping change score Week 12-Week 4 (min/16 h awake) I vs. C: 6.6, 95% CI 2.5,10.7, $p = 0.002$ vs. −0.2, 95% CI −4.6, 4.2, $p = 0.9$, MD (I–C) 6.9, 95% CI 0.8, 12.9, $p = 0.03$; Mean time moderate-vigorous stepping change score Week 12-Week 4 (min/16 h awake) I vs. C: −4.8, 95% CI −12.5,2.9, p	Exercise sessions: 86% ($n = 31$) patients attended all four sessions, 1 AE was recorded (a patient fell during a lunge exercise, no injury sustained).<br><br>During the initial tailoring session, intervention patients selected two "sitting danger zones" to target. Most commonly identified zones were: watching television (12/36), computer work (11/36), and reading (8/36). Each patient also set an MVPA goal (which may have included more than one type of activity). Most commonly identified goals were: brisk walking (12/18), cycling (5/18), and going to the gym (4/18).<br><br>All intervention patients attended both tailoring sessions and received text messages for the first 4 weeks of the program, however 4 participants opted out from receiving texts for the last 8 weeks (reasons included: $n = 1$ sufficiently self-motivated to continue without texts, $n = 1$ not finding texts useful, $n = 1$ overseas travel, $n = 1$ not liking the directive language of the texts). Intervention patients opted to receive an average of 8 messages/fortnight (range 6–12, possible range 6–17). 13/18 opted to receive the minimum amount of prompts for sedentary behaviour, 12/18 opted for the minimum amount of prompts for MVPA. The average reply rate to the goal checks was 78% (8/18 patients replied to all goal checks; 83 replies, of which 8 were screened by the study staff and the remainder were automatically screened).

	= 0.2 vs. −6.6, 95% CI −14.7, 1.4, p = 0.1, MD (I–C) 1.9, 95% CI −9.2, 13.0, p = 0.7<br><br>MULTIMEDIA ACTIVITY RECALL FOR CHILDREN AND ADULTS (INTENSITY) OUTCOMES:<br>Sample sizes: I group n = 18 at Week 4, n = 15 at Week 12; C group n = 17 at Week 4, n = 15 at Week 12<br>Mean out of clinic time use for sleep change score Week 12-Week 4 (min/day) I vs. C: 34.1, 95% CI 3.0, 65.2, p = 0.03 vs. −29.9, 95% CI −61.2, 1.4, p = 0.06, MD (I–C) 64.0, 95% CI 19.9, 108.2, p =0.004; Mean out of clinic time use for sitting change score Week 12-Week 4 (min/day) I vs. C: −36.2, 95% CI −79.2, 6.9, p = 0.1 vs. 24.8, 95% CI −18.4, 68, p =0.3, MD (I–C) −61.0, 95% CI −121.9, 0.0, p = 0.05; Mean out of clinic time use for light PA change score Week 12-Week 4 (min/day) I vs. C: −5.2, 95% CI −41.8, 31.4, p = 0.8 vs. 13.4, 95% CI -23.8, 50.6, p = 0.5, MD (I–C) −18.6, 95% CI −70.8, 33.6, p = 0.5; Mean out of clinic time use for moderate PA change score Week 12-Week 4 (min/day) I vs. C: −5.7, 95% CI −42.1, 30.7, p = 0.8 vs. −15.7, 95% CI -52.7, 21.3, p = 0.4, MD (I–C) 10.0, 95% CI −41.8, 61.8, p = 0.7; Mean out of clinic time use for vigorous PA change score Week 12-Week 4 (min/day) I vs. C: 8.2, 95% CI −9.2,25.6, p = 0.4 vs. 5.0, 95% CI -12.4, 22.4, p = 0.6, MD (I–C) 3.2, 95% CI −21.4, 27.8, p = 0.8; Mean out of clinic time use for moderate-to-vigorous PA change score Week 12-Week 4 (min/day) I vs. C: 4.1, 95% CI -27.0, 35.2, p = 0.8 vs. −10.0, 95% CI -41.5, 21.5, p = 0.5, MD (I–C) 14.1, 95% CI −30.1, 58.3, p = 0.5	Patient satisfaction questionnaire among intervention group, mean scores (n = 17): satisfaction with tailoring sessions and text messages was 4.5 SD 0.6 and 4.1 SD 1.1 (scores ranged from 1–5), 71% extremely satisfied with tailoring sessions and 53% with text messages, 59% found tailoring sessions and 47% found text messages extremely useful for meeting PA goal, 47% found tailoring sessions and 47% found text messages extremely useful for meeting sitting goal, 100% read the text messages, 88% found it very easy and 12% found it easy to understand the text messages<br><br>Patient response to request for program suggestions (n = 14): n = 9 recommended the program without suggestions or complaint, n = 2 indicated the texts had not succeeded in prompting a sense of motivation (sometimes prompting guilt), n = 1 indicated that the text content was incongruent with what was happening when it was received, n = 1 wanted more texts that required a response, n = 1 suggested access to online exercise prescription

MULTIMEDIA ACTIVITY RECALL FOR CHILDREN AND ADULTS (DOMAINS) OUTCOMES:

Sample sizes: I group $n = 18$ at Week 4, $n = 15$ at Week 12; C group $n = 17$ at Week 4, $n = 15$ at Week 12

Mean out of clinic time use for chores change score Week 12-Week 4 (min/day) I vs. C: 10.4, 95% CI −29.9, 50.7, $p = 0.6$ vs. −7.1, 95% CI −47.7, 33.6, $p = 0.7$, MD (I–C) 17.4, 95% CI −39.8, 74.6, $p = 0.6$; Mean out of clinic time use for cultural change score Week 12-Week 4 (min/day) I vs. C: −4.5, 95% CI −14.7, 5.6, $p = 0.4$ vs. −2.7, 95% CI −13.2, 7.8, $p = 0.6$, MD (I–C) −1.8, 95% CI −16.4, 12.8, $p = 0.8$; Mean out of clinic time use for PA change score Week 12-Week 4 (min/day) I vs. C: 5.8, 95% CI −5.2, 16.7, $p = 0.3$ vs. 9.0, 95% CI −1.9, 19.9, $p = 0.1$, MD (I–C) −3.2, 95% CI −18.6, 12.2, $p = 0.7$; Mean out of clinic time use for quiet time change score Week 12-Week 4 (min/day) I vs. C: −20.6, 95% CI −56.1, 14.9, $p = 0.3$ vs. 20.7, 95% CI −15.1, 56.5, $p = 0.3$, MD (I–C) −41.3, 95% CI −91.8, 9.1, $p = 0.1$; Mean out of clinic time use for screen time change score Week 12-Week 4 (min/day) I vs. C: −1.7, 95% CI −70.8, 67.3, $p = 1$ vs. −13.7, 95% CI −61.5, 34.2, $p = 0.6$, MD (I–C) −48.3, 95% CI −115.9, 19.3, $p = 0.2$; Mean out of clinic time use for self-care change score Week 12-Week 4 (min/day) I vs. C: -1.1, 95% CI −19.3, 17.0, $p = 0.9$ vs. 5.7, 95% CI −12.6, 24.1, $p = 0.5$, MD (I–C) −6.9, 95% CI −32.7, 18.9, $p = 0.6$; Mean out of clinic time use for social change score Week 12-Week 4 (min/day) I vs. C:18.4, 95% CI −22.0, 58.8, $p = 0.4$ vs. 18.7, 95% CI −18.6, 56.0, $p = 0.3$, MD (I–C) 4.0, 95% CI −48.7, 56.7, $p = 0.9$; Mean out of

	clinic time use for transport change score Week 12-Week 4 (min/day) I vs. C: 18.2, 95% CI −15.8, 52.2, $p = 0.3$ vs. −3.4, 95% CI −37.9, 31.1, $p = 0.8$, MD (I–C) 21.6, 95% CI −26.9, 70.0, $p = 0.4$; Mean out of clinic time use for work and study change score Week 12-Week 4 (min/day) I vs. C: 0.2, 95% CI −25.1, 25.4, $p = 1$ vs. 1.9, 95% CI -23.5, 27.3, $p = 0.9$, MD (I–C) −1.7, 95% CI −37.5, 34.0, $p = 0.9$	
Haggerty, 2017 [28]	Median SF-12 physical health component 6-month change score I vs. C1 vs. C2: 0.9 IQR −0.7, 4.8 ($n = 11$) vs. 5.4 IQR 3.8, 15.0 ($n = 11$) vs. 7.4 IQR 1.8, 11.0 ($n = 10$); $p = 0.04$ between I and C1<br><br>Median weight 6-month change score (kilograms) I vs. C1 vs. C2: −4.4 IQR −7.9, 1.1, −3.9% of total weight loss ($n = 11$) vs. −3.0 IQR −11.5, −0.1, −4.6% of total weight loss ($n = 11$) vs. −1.8 IQR −5.2, −0.5, −3.3% of total weight loss ($n = 10$), $p > 0.05$; Median waist circumference 6-month change score (centimeters) I vs. C1 vs. C2: −5.9 IQR −10.5, 2.6 ($n = 11$) vs. −3.7 IQR −7.6, 3.0 ($n = 11$) vs. −4.0(IQR −13.2, 0.5 ($n = 10$), $p > 0.05$<br><br>Median total PA 6-month change score (METs/week) I vs. C1 vs. C2: 588.0 IQR 88.0, 931.2 ($n = 11$) vs. 175.5 IQR −343.5, 348.5 ($n = 11$) vs. 1454.5 IQR 619.9, 2655.4 ($n = 10$), $p < 0.05$ between C1 and C2; Median vigorous PA 6-month change score (METs/week) I vs. C1 vs. C2: 0 IQR 0.0, 480.0 ($n = 11$) vs. 0 IQR 0, 0 ($n = 11$) vs. 1120.0 IQR 0.0, 1840.0 ($n = 10$), $p = 0.008$ between I and C2, $p = 0.034$ between C1 and C2; Median walking activity 6-month change score (METs/week) I vs. C1 vs. C2: 430.7 IQR 132.0, 594.0 ($n = 11$) vs.	

		41.3 IQR −280.5, 148.5 (n = 11) vs. 24.8 IQR -198.0, 429.0 (n = 10), p = 0.02 between I and C1<br><br>Median Multidimensional Body Self Relations Questionnaire - Appearance subscale 6-month change score I vs. C1 vs. C2: 0.0 IQR −1.0,0.0 (n = 11) vs. −3.5 IQR -5.0, −1.0 (n = 11) vs. −0.5 IQR 1.5, 0 (n = 10); p = 0.04 between I and C1<br><br>Median Female Sexual Function Index 6-month change score I vs. C1 vs. C2: 0.0 IQR 0.0, 1.2 (n = 11) vs. 0.4 IQR 0.4, 0.8 (n = 11) vs. 0.0 IQR 0.0, 0.0 (n = 10); p = 0.03 between C1 and C2	
Hershman, 2020 [29]			Medication adherence failure (based on urine samples, accounting for censoring) in I vs. C: total 283 vs. 303 events, HR 0.9, 95% CI 0.8–1.05, p = 0.2 (year 1 - 50.9% vs. 57.2%, year 2 - 70.4% vs. 74.4%, year 3 - 81.9% vs. 85.6%). Patient-reported medication adherence failure in I vs. C: HR 1.2, 95% CI 0.7–2.0, p = 0.57 (year 3 - 10.4% vs. 10.3%). Site-reported medication adherence failure in I vs. C: HR 1.3, 95% CI 0.9–2.01, p = 0.2 (year 3 - 21.9% vs. 18.9%).<br><br>Note: reported several other medication adherence failure sensitivity analyses (see paper for details).
Rico, 2020 [30]	Number of patients experiencing side effects in cycle 1 in I vs. C: 0–3 side effects 26 vs. 17, 4–14 side effects 28 vs. 42, p = 0.05; Number of patients experiencing side effects in cycle 2 in I vs. C: 0–3 side effects 17 vs. 14, 4–14 side effects 29 vs. 38, p =		On average 52 SMS messages were sent from day 1 to beginning of cycle 4, all were sent in the morning and were sent automatically cHEmotHErApp. All patients reported to having read the daily texts.<br><br>Patients receiving text messages considered them helpful to cope with treatment, 72.1% reported being very satisfied, 27.9% reported being satisfied. Regarding followed guidelines from text messages received 65.1%

0.4; Number of patients experiencing side effects in cycle 3 in I vs. C: 0–3 side effects 19 vs. 15, 4–14 side effects 24 vs. 29, $p = 0.4$<br><br>Number of patients experiencing changes in skin in I vs. C cycle 1: 11 vs. 16, $p = 0.5$; cycle 2: 16 vs. 19, $p = 1$; cycle 3: 15 vs. 20, $p = 0.4$<br><br>Number of patients experiencing changes in sense of taste in I vs. C cycle 1: 18 vs. 29 $p = 0.06$; cycle 2: 20 vs. 30, $p = 0.2$; cycle 3: 26 vs. 26, $p = 1$<br><br>Number of patients experiencing fatigue in I vs. C cycle 1: 26 vs. 34, $p = 1$; cycle 2: 25 vs. 32 $p = 0.5$; cycle 3: 25 vs. 27, $p = 0.8$<br><br>Number of patients experiencing diarrhea in I vs. C cycle 1: 11 vs. 13, $p = 1$; cycle 2: 9 vs. 8, $p = 0.6$; cycle 3: 10 vs. 12, $p = 0.8$<br><br>Number of patients experiencing pain in I vs. C cycle 1: 21 vs. 31, $p = 0.2$; cycle 2:		reported to have followed all text messages, 32.6% reported to have followed almost all text messages, 2.3% patients reported to have followed half.<br><br>The side effects most observed in the first 3 cycles of treatment in C was nausea (73%), fatigue (60%), and changes in the sense of taste (57%). In I group fatigue (53%), weakness (49%) and nausea (49%).

	21 vs. 28, *p* = 0.5; cycle 3: 20 vs. 20, *p* = 1<br><br>Number of patients experiencing lack of appetite in I vs. C cycle 1: 21 vs. 25, *p* = 0.7; cycle 2: 21 vs. 23, *p* = 1; cycle 3: 16 vs. 14, *p* = 0.8<br><br>Number of patients experiencing shortness of breath in I vs. C cycle 1: 9 vs. 14, *p* = 0.5; cycle 2: 8 vs. 12, *p* = 0.6; cycle 3: 5 vs. 6, *p* = 1<br><br>Number of patients experiencing fever in I vs. C cycle 1: 2 vs. 7, *p* = 0.2; cycle 2: 4 vs. 4, *p* = 1; cycle 3: 1 vs. 5, *p* = 0.2<br><br>Number of patients experiencing Mouth Lesions in I vs. C cycle 1:16 vs. 14, *p* = 0.5; cycle 2: 12 vs. 11, *p* = 0.6; cycle 3: 8 vs. 11, *p* = 0.6<br><br>Number of patients experiencing weakness in I vs. C cycle 1: 22 vs. 29, *p* = 0.5; cycle 2: 24 vs. 23, *p* = 0.5; cycle 3: 25 vs. 23, *p* = 0.7<br><br>Number of patients experiencing indigestion in I vs. C	

cycle 1: 11 vs. 22, $p < 0.05$; cycle 2: 10 vs. 18, $p = 0.2$; cycle 3: 14 vs. 18, $p = 0.5$

Number of patients experiencing constipation in I vs. C cycle 1: 26 vs. 26, $p = 0.7$; cycle 2: 15 vs. 18, $p = 1$; cycle 3: 14 vs. 15, $p = 1$

Number of patients experiencing nausea in I vs. C cycle 1: 27 vs. 41, $p = 0.03$; cycle 2: 20 vs. 40, $p = 0.001$; cycle 3: 24 vs. 33, $p = 0.06$

Number of patients experiencing vomiting in I vs. C cycle 1:12 vs. 17, $p = 0.5$; cycle 2: 8 vs. 15, $p = 0.2$; cycle 3: 7 vs. 10, $p = 0.6$

Mean total sum of experienced side effects cycle 1/2 in I vs. C: 20.1 SD 0.8/21.5 SD 0.8, $p = 0.03$ vs. 24.9 SD 1.0/23.9 SD 0.9, $p = 0.3$; Mean total sum of experienced side effects cycle 2/3 in I vs. C: 21.3 SD 0.9/23.3 SD 1.2, $p = 0.02$ vs. 23.6 SD 1.0/23.7 SD 0.9, $p = 0.9$; Mean total sum of experienced side effects cycle 1/3 in I vs. C: 20.1 SD 0.8/23.3

	SD 1.2, *p* = 0.001 vs. 25.1 SD 1.1/23.7 SD 0.9, *p* = 0.3		
Spoelstra, 2016 [34]	Mean Total number of symptoms in I vs. C: 4.9 SD 0.4 vs. 5.2 SD 0.6, *p* = 0.71 (ES 0.09);<br>Mean summed severity in I vs. C: 23.0 SD 2.7 vs. 26.5 SD 3.7, *p* = 0.45 (ES 0.2);<br>Mean summed interference in I vs. C: 18.2 SD 2.7 vs. 21.9 SD 3.7, *p* = 0.41 (ES 0.2)	Mean PROMIS Depression score in I vs. C: 44.6 SD 1.0 vs. 44.2 SD 1.3, *p* = 0.8 (ES 0.06); Mean PROMIS Physical function score in I vs. C: 45.7 SD 0.9 vs. 45.7 SD 1.3, *p* = 1 (ES 0)<br><br>Mean Cognitive function - Effective action subscale score in I vs. C: 49.7 SD 1.5 vs. 53.4 SD 2.0, *p* = 0.2 (ES 0.4);<br>Mean Cognitive function - Attentional lapses subscale score in I vs. C: 23.5 SD 0.7 vs. 24.1 SD 0.9, *p* = 0.6 (ES 0.2); Mean Cognitive function -Interpersonal effectiveness subscale score in I vs. C: 22.1 SD 0.7 vs. 23.7 SD 0.9, *p* = 0.2 (ES 0.4)<br><br>Mean Social Support score in I vs. C: 3.7 SD 0.4 vs. 2.4 SD 0.5, *p* = 0.04 (ES 0.5)	Mean adherence to OA in I vs. C: 6.5 SD 0.4 vs. 7.2 SD 0.5, *p* = 0.26 (ES 0.29); Mean<br>MASES-R score in I vs. C: 4.6 SD 3.4 vs. 4.8 SD 3.2, *p* = 0.78<br><br>Satisfaction survey (*n* = 39): 92% reported satisfaction (very much/highly satisfied) and 97.4% would recommend texts as a way to help patients remember OAs. 100% would recommend to their oncologist to monitor adherence. 89.2% found the texts mostly helpful, 73.7% thought texts helped remind patients to take OAs on time, 78.9% read all texts sent.<br><br>5.3% encountered problems with the text message system, these participants were between the ages of 51+, poor agreement of patient self-reported dose changes and medical reported ones was seen.
Spoelstra, 2015 [33]	Mean total number of symptoms in I vs. C: 3.9 SD 0.5 vs. 5.3 SD 0.46, *p* = 0.04 (ES 0.5); Mean summed severity in I vs. C: 22.7 SD 3.0 vs. 24.4 SD 2.6, *p* = 0.7 (ES 0.1); Mean summed interference in I vs. C: 17.1 SD 2.3 vs. 18.8 SD 2.0, *p* = 0.6 (ES 0.1)	Mean PROMIS Depression score in I vs. C: 44.7 SD 1.3 vs. 44.9 SD 1.2, *p* = 0.9 (ES 0.03); Mean PROMIS Physical function score in I vs. C: 47.6 SD 1.2 vs. 44.9 SD 1.09, *p* = 0.1 (ES 0.4)<br><br>Mean Cognitive function - Effective action subscale score in I vs. C: 49.8 SD 1.9 vs. 51.5 SD 1.7, *p* = 0.5 (ES 0.2); Mean Cognitive function - Attentional lapses subscale score in I vs. C: 23.6 SD 1.04 vs. 24.04 SD 0.9, *p* = 0.8 (ES 0.07); Mean Cognitive function - Interpersonal Effectiveness subscale score in I vs. C: 22.6 SD 0.8 vs. 23.5 SD 0.7, *p* = 0.4 (ES 0.2)	Mean Medication adherence rating scale score in I vs. C: 0.7 SD 0.2 vs. 0.6 SD 0.2, *p* = 0.8 (ES 0.07); Mean Medication adherence self-efficacy scale score in I vs. C: 30.7 SD 0.3 vs. 31.2 SD 0.3, *p* = 0.2 (ES 0.3); Overall mean adherence in I vs. C: 6.0 SD 0.5 vs. 6.0 SD 0.5, *p* = 1 (ES 0); Mean relative dose intensity (RDI) in I vs. C: 1.1 SD 0.1 vs. 0.7 SD 0.2, *p* = 0.1 (ES 0.6)<br><br>All were somewhat (*n* = 2) or highly (*n* = 35) satisfied with their participation in the study. 34/36 found the text messages mostly helpful and 28/35 agreed it helped them take their medication on time. All but 1 person was satisfied with the texts and 30/35 read all the texts, 32/37 would recommend as a system to re-

	Mean Brief Medical Questionnaire score in I vs. C: 26.3 SD 0.8 vs. 26.6 SD 0.7, *p* = 0.8 (ES 0.07)<br><br>Mean Medication specific social support scale score in I vs. C: 3.5 SD 0.5 vs. 3.03 SD 0.4, *p* = 0.4 (ES 0.2)	mind patients to take OAs and 34/37 would recommend as a way to monitor adherence, 29/36 would recommend to a family member or friend and 27 would recommend to clinician.<br><br>7/37 encountered a problem with automated voice recordings, 1/36 encountered a problem with texts<br><br>1359 texts were sent to patients (1111 adherence, 116 symptom management texts, 52 additional week texts, 53 welcome and 17 end of study), 39/40 participants completed entire text intervention
Tan, 2020 [31]		Patients adherent according to SMAQ in I vs. C vs. All: 52.0% vs. 54.6% vs. 53.3%; 6-month SMAQ in I vs. C: 72.4% vs. 59.5%, *p* = 0.03, OR 1.8, 95% CI 1.04, 3.05; 1-year<br>SMAQ in I vs. C: 68.9% vs. 65.8%, *p* = 0.6, OR 1.2, 95% CI 0.7, 2.0;<br>SMAQ over 1 year period in I vs. C: 71.0% vs. 61.6%, *p* < 0.05, OR 2.4, 95% CI 1.01, 5.5<br><br>Median androstenedione in I vs. C vs. All: 2.8 IQR 2.2–3.5 vs. 2.9 IQR 2.0–3.7 vs. 2.9 IQR 2.1–3.6; Mean z-score of estrone in I vs. C vs. All: 2.0 vs. 1.9 vs. 1.9; Patients with Estradiol ECLIA less than 18.4 pmol/L in I vs. C vs. All: 100% vs. 98.1% vs. 99.1%; Patients with Estradiol ECLIA greater than 18.4pmol/L in I vs. C vs. All: 0% vs. 1.9% vs. 0.9%<br><br>Model adjusting for baseline hormone levels, I vs. C *n* = 12 vs. *n* = 13:<br>Intervention had no significant effect on androstenedione levels (mean z-score in I vs. C: 2.88 vs. 2.88, effect estimate 1.00, 95% CI 0.91, 1.10, *p* = 0.978); Intervention had no significant effect on estrone levels (mean z-

		score in I vs. C: 3.14 vs. 2.18, effect estimate 0.96, 95% CI –0.44, 2.36, *p* = 0.178); Intervention had no significant effect on estradiol levels (mean z-score in I vs. C: 94.8 vs. 97.6, effect estimate 0.42, 95% CI 0.08,2.24, *p* = 0.312)<br><br>Overall most patients agreed that SMS was easy to understand (99.2%) with acceptable timing of reminder 98.4%. 96.7% agreed that there was enough information provided in the SMS reminder. 2 patients (1.6% suggested using less words or emojis and changing the template to avoid repetition. Frequency of reminder was largely accepted at 87.7%. 2 patients (1.6%) suggested customizing the schedule according to their medication routine. 13.9% suggested increased frequency. 86.1% would recommend this service as part of routine care. 78.7% agreed they were useful, those who did not find them useful were already using reminders however they recognized who it would be useful to those without reminders already. At baseline, 20.9% of the patients had a medication reminder. According to SMAQ, 53.3% of patients were adherent to AET at baseline
Villaron, 2018 [32]	Reduced activity (mean MFI-20 score) at Week 7 in I vs. C: 8.6 SD 3.3 vs. 12.4 SD 4.8, $p < 0.01$; Reduced activity (mean MFI-20 score) at Week 8 in I vs. C: 8.2 SD 4.1 vs. 11.8 SD 4.1, $p < 0.01$; Reduced motivation (mean MFI-20 score) at Week 7 in I vs. C: 7.3 SD 3.8 vs. 11.1 SD 4.7, $p < 0.01$; Reduced motivation (mean MFI-20 score) at Week 8 in I vs. C: 7.9 SD 3.7 vs. 10.7 SD 3.9, $p < 0.05$; Mental Fatigue (mean MFI-20 score) at Week 7 in I vs. C: 6.7 SD 3.4 vs. 10.5 SD 4.3, $p < 0.01$; Mental Fatigue (mean MFI-20 score) at Week 8 in I vs. C: 6.9 SD 3.8 vs. 10.0 SD 4.2, *p* <	Compliance of surveys was 64.58%, 71% of patients participated convincingly in the study.

	0.05; Physical Fatigue (mean MFI-20 score) at Week 7 in I vs. C: 9.3 SD 3.9 vs. 12.7 SD 4.9, $p <$ 0.01; Physical Fatigue (mean MFI-20 score) at Week 8 in I vs. C: 9.8 SD 4.6 vs. 12.3 SD 4.6, $p <$ 0.05<br><br>Role function (mean QLQ-30 score) at Week 7 in I vs. C: 83.3 SD 18.7 vs. 60.7 SD 28.9, $p < 0.01$; Role function (mean QLQ-30 score) at Week 8 in I vs. C: 82.3 SD 18.8 vs. 63.6 SD 23.1, $p < 0.01$; Physical capacity (mean QLQ-30 score) at Week 7 in I vs. C: 88.3 SD 13.5 vs. 75.5 SD 18.9, $p < 0.01$; Physical capacity (mean QLQ-30 score) at Week 8 in I vs. C: 88.2 SD 13.6 vs. 83.6 SD 12.7, $p = 0.3$<br><br>Mean steps at Week 1 in I vs. C: 9249.6 SD 5939 vs. 7323.4 SD 4545.7; Mean steps at Week 2 in I vs. C: 7382.3 SD 3798.4 vs. 8835.5 SD 8374.2; Mean steps at Week 3 in I vs. C: 7746.2 SD 4233.8 vs. 8209.8 SD 9574.9; Mean steps at Week 4 in I vs. C: 7016.7 SD 3895.4 vs. 9072.3 SD 6249.3; Mean steps at Week 5 in I vs. C: 7643.3 SD 3147.2 vs. 7933.3 SD 5183.7; Mean steps at Week 6 in I vs. C: 5861.1 SD 2539.7 vs. 6770.5 SD 4293.7; Mean steps at Week 7 in I vs. C: 6408.8 SD 2942.8 vs. 6440.2 SD 4881.2; Mean steps at Week 8 in I vs. C: 6782.6 SD 2805.2 vs. 6921.3 SD 4304.8	
	Non-Randomized Interventional/Observational Studies	
Bade, 2018 [35]	Mean daily step count at Week 0 vs. Week 3: I group ($n = 15$) 4906.1 SD 256.8 vs. 5241.2 SD 291.7 (ES 0.02); C group ($n = 22$) 5128.2 SD 223.7 vs. 5247.2 SD 242.9 (ES 0.05); after adjusting for first week average, gender, and age, the average weekly change increased for both groups.	I vs. C: subjects never using the device: 0% vs. 21% (out of $n = 15$ vs. $n = 29$); days no step counts were collected: 11% vs. 38% (out of $n = 420$ vs. $n = 812$).

			Feedback survey among intervention group: 92% found intervention helpful (out of n = 13), 75% intended to continue tracking activity after the study (out of n = 12), 85% would participate in another PA study (out of n = 13), 83% not interested in group activities (out of n = 12), preferred frequency for text messages (out of n = 12): 50% said 1/day, 17% said 2/day, 33% said 3/day, preferred time for text messages (out of n = 12): 17% said morning, 58% said noon, 25% said afternoon.
Chow, 2019 [36]		Mean PHQ-4 score: 1.73 SD 2.3 (n = 9 reported at least a moderate level of distress ≥6 at any point; for the anxiety subscale n = 14 reported scores above the recommended cut-off ≥ 3 at any point; for the depression subscale n = 11 reported scores above the recommended cut-off ≥ 3 at any point).<br><br>For each patient, screeners were completed within a range of 0–1119.3 miles of each other. Across all patients, the median distance between screener locations was 79.1 miles.<br><br>Analyses of model fit indicated significant heterogeneity in variability of distress scores over time and across patients.	Screener adherence rate was 75%; of the n = 44 who completed at least one screener, 7% completed 1, 2% completed 2, 23% completed 3, and 68% completed 4; mean time to complete was 75 SD 58 seconds for the first, 50 SD 42 seconds for the second, 43 SD 25 seconds for the third, and 53 SD 55 seconds for the fourth.<br><br>Feasibility questionnaire mean scores: overall the text messages did a good job of capturing my weekly mood 5.9/7 (SD 1.6), I was worried about my privacy in responding to the text messages 1.9/7 (SD 2.0), I would be willing to continue responding to the same weekly messages throughout my cancer care 6.3/7 (SD 1.7), it would be OK if my care providers saw my weekly message responses so they could respond to my distress levels and needs 6.7/7 (SD 1.2), being able to communicate my distress levels and needs through my smartphone meets an important need 5.7/7 (SD 1.8), remotely monitoring my distress levels and needs throughout my cancer treatment makes me feel better cared for 6.1/7 (SD 1.3), the timing and duration of text messages were a significant burden 1.5/7 (SD 1.7), I would be willing to wear a smartwatch if it improved the ability of my care providers to understand my distress levels and needs 5.6/7 (SD 2.1), it would be useful

			for me to see my distress levels and needs from previous weeks 4.6/7 (SD 2.1).<br><br>Mean Usability, Satisfaction, and Ease of use scale score was 6.9/7 for ease of use, 6.9/7 (SD 0.4) for ease of learning, and 6.5/7 (SD 0.3) for satisfaction.
Krok- Schoen, 2019 [11]	Mean Breast Cancer Prevention Trial Symptom Checklist score I vs. C (n = 37): 0.8 SD 0.5 vs. 0.7 SD 0.5, MD (I–C) 0.04, (95% CI −0.06, 0.1), $p = 0.4$<br><br>Mean Brief Pain Inventory score I vs. C (n = 11): 3.6 SD 2.6 vs. 2.8 SD 2.8, MD (I–C) 0.8 (95% CI −0.4, 2.02), $p = 0.2$<br><br>Mean Fatigue Symptom Inventory score I vs. C (n = 36): 1.6 SD 2.0 vs. 1.8 SD 2.2, MD (I–C) −0.3 (95% CI -0.8, 0.3), $p = 0.3$	Mean CES-D score I vs. C (n = 31): 5.7 SD 8.0 vs. 6.9 SD 9.1, MD (I–C) −1.2 (95% CI −3.5, 1.01), $p = 0.3$<br><br>Mean SF-8 mental health component score I vs. C (n = 36): 53.0 SD 6.5 vs. 49.9 SD 7.8, MD (I–C) 3.03 (95% CI 0.9, 5.2), $p = 0.007$; Mean SF-8 physical health component score I vs. C (n = 36): 46.4 SD 10.6 vs. 45.4 SD 10.3, MD (I–C) 1.0 (95 % CI −1.7, 3.6), $p = 0.5$<br><br>Mean Perceived Stress Scale score I vs. C (n = 36): 15.6 SD 8.24 vs. 17.1 SD 9.39, MD (I–C) −1.5 (95% CI −2.9, −0.07), $p = 0.04$<br><br>Mean Social Desirability Response score I vs. C (n = 37): 21.4 SD 2.2 vs. 21.6 SD 1.8, MD (I–C) −0.2 (95% CI −0.99, 0.6), $p = 0.6$<br><br>Mean Medical Outcomes Study Social Support score I vs. C (n = 36): 90.9 SD 9.4 vs. 89.6 SD 10.2, MD (I–C) 1.4 (95% CI −0.7, 3.4), $p = 0.2$<br><br>Median estradiol I vs. C: 1.5 (n = 32) vs. 5.1 (n = 38), $p < 0.0001$; Median estrogen I vs. C: −1.0 (n = 30) vs. 19.7 (n = 38), $p < 0.0001$; Median estone I vs. C: −1.0 (n = 30) vs. 15.2 (n = 38), $p < 0.0001$	12/39 did not complete the intervention for the following reasons: being busy, not feeling well, or forgetfulness.<br><br>Mean Morisky Adherence score I vs. C (n = 36): 1.2 SD 1.3 vs.1.9 SD 1.7, MD (I–C) −0.8 (95% CI −1.4, −0.2), $p = 0.02$<br><br>Patient post-intervention feasibility questionnaire (n = 37): 97.3% reported a positive experience, >91% believed that they benefited from study participation, 81% agreed or strongly agreed that the daily reminder messages helped them to be more adherent to their medication, 95% agreed or strongly agreed that the intervention would be helpful for future patients taking the medication, 97.3% agreed or strongly agreed that the instructions for the text messaging system were helpful.<br><br>Physician post-intervention feasibility questionnaire (n = 7): All agreed or strongly agreed that their patients were helped by participating in the study, >85% agreed or strongly agreed that (1) the video positively influenced their patient's communication, (2) their patient valued participation in the study, and (3) being informed about their patient's adherence helped them provide better care, all agreed or strongly agreed that they would recommend that patients use this intervention when taking AHT medication.

		Mean Concerns About Recurrence score I vs. C (n = 37): 9.7 SD 5.0 vs. 10.9 SD 6.6, MD (I–C) −1.1 (95% CI −2.3, 0.04), p = 0.06<br><br>Mean Communication and Attitudinal Self-Efficacy score I vs. C (n = 36): 27.8 SD 4.4 vs. 27.8 SD 3.2, MD (I–C) −0.06 (95% CI -1.7, 1.6), p = 0.9	
Maguire, 2015 [37]	Median ESAS pain score post-treatment vs. baseline (n = 16): 4 range 0–8 vs. 1.5 range 0–7;<br>Median ESAS tiredness score post-treatment vs. baseline (n = 16): 4 range 0–9 vs. 5 range 1–8; Median ESAS nausea score post-treatment vs. baseline (n = 16): 2 range 0–6 vs. 0 range 0–8; Median ESAS appetite score post-treatment vs. baseline (n = 16): 4 range 0–10 vs. 5 range 0–10; Median ESAS drowsiness score post-treatment vs. baseline (n = 16): 3 range 0–8 vs. 3.5 range 0–9; Median ESAS breathlessness score post-treatment vs. baseline (n = 16): 3 range 0–9 vs. 3.5 range 0–9; Median ESAS other score post-treatment vs. baseline (n = 16): 0 range 0–7 vs. 0 range 0–6	Median ESAS depression score post-treatment vs. baseline (n = 16): 0 range 0–8 vs. 0 range 0–8; Median ESAS anxiety score post-treatment vs. baseline (n = 16): 1 range 0-8 vs. 0.5 range 0–10; Median ESAS well-being score post-treatment vs. baseline (n = 16): 4.5 range 0–7 vs. 4.5 range 0–10<br><br>Mean STAI-Y state score post-treatment vs. baseline (n = 16): 46.4 SD 5.1 vs. 43.9 SD 9.0; Mean STAI-Y trait score post-treatment vs. baseline (n = 16): 44.9 SD 5.3 vs. 43.0 SD 8.3<br><br>Mean SUPPH29 Positive attitude score post-treatment vs. baseline (n = 16): 56.1 SD 14.6 vs. 48.1 SD 16.1; Mean SUPPH 29 Stress score post-treatment vs. baseline (n = 16): 26.6 SD 9.4 vs. 27.4 SD 10.9; Mean SUPPH 29 making decisions score post-treatment vs. baseline (n = 16): 9.4 SD 3.7 vs. 8.4 SD 4.0<br><br>Mean FACT-L physical wellbeing score post-treatment vs. baseline (n = 16): 17.4 SD 6.2 vs. 19.7 SD 6.5; Mean FACT-L social/family wellbeing score post-treatment vs. baseline (n = 16): 17.1 SD 4.0 vs. 18.6 SD 4.0; Mean FACT-L emotional wellbeing score post-treatment vs. baseline (n = 16): 16.5 SD 6.6 vs. 16.6 SD 6.4; Mean	Over 12 months 182 alerts were generated (138 amber, 44 red)<br><br>78% felt the questionnaire covered all relevant topics. All agreed the handset helped them manage symptoms and communicate with the physician/nurses. Patients expressed feelings of reassurance offered by ASyMS-R due to rapid feedback of healthcare professionals and it reduced the uncertainty experienced by the patients, particularly at times when they were at home and were unsure as to whether they should contact health professionals or not. 89% perceived self-care advice offered as easy to understand and user friendly. 6 patients admitted to never or only sometimes reading self-care information (either during or after treatment). Some commented on the lack of training on the self-care component others said they did not use this as they had already received similar information from healthcare professionals.<br><br>Response rate of 28.1%. Reasons for refusal included poor health status, patients feeling that they were being adequately managed by their clinical team and therefore perceived no need for additional supportive care interventions, and lack of familiarity with the use of technology. 5 patients died before post study assessment. 9 patients indicated they had not received enough training on the use of ASyMS-R handset, 78%

		FACT-L functional wellbeing score post-treatment vs. baseline ($n = 16$): 14.6 SD 6.6 vs. 12.0 SD 7.8; Mean FACT-L lung cancer subscale score post-treatment vs. baseline ($n = 16$): 19.6 SD 7.1 vs. 21.2 SD 6.7; Mean FACT-L total score post-treatment vs. baseline ($n = 16$): 85.1 SD 21.6 vs. 86.2 SD 24.7; Mean FACT-L trial outcome index score post-treatment vs. baseline ($n = 16$): 51.6 SD 15.5 vs. 52.1 SD 17.8	felt the questionnaire covered all relevant topics. All agreed the handset helped them manage symptoms and communicate with the physician/nurses.<br><br>All encountered no or rare problems using the handset (100%) and most answering submitting questionnaires (90%), reading the self-care information after submitting a questionnaire or again at a later data (100%) or finding cancer info pages (89%). 6 patients admitted to never or only sometimes having read the self-care information pages, either during or after treatment. In the post study interviews, some of the participants commented on how they were not trained on using this component of the system. Others commented on how they did not use the self-care information because of having received similar information from their healthcare professionals.
Mougalian, 2017 [38]	Number of patients with any symptom Tamoxifen vs. AI vs. all: 35 vs. 56 ($p = 0.2$) vs. 91; Number of patients with hot flashes Tamoxifen vs. AI vs. all: 29 vs. 42 ($p = 0.2$) vs. 71; Number of patients with joint aches/pains Tamoxifen vs. AI vs. all: 7 vs. 45 ($p < 0.01$) vs. 52; Number of patients with vaginal symptoms Tamoxifen vs. AI vs. all: 16 vs. 18 ($p = 0.1$) vs. 34; Number of patients with other symptoms Tamoxifen vs. AI vs. all: 24 vs. 37 ($p = 0.5$) vs. 61; Number of patients		Mean of 21 texts per week were received by patients, 86.1% of patients responded to all of daily texts, among those who completed the pilot the response rate was 92.2%. The overall adherence rate was 85.1%. Of those who completed the pilot 93.3% reported taking 80+% of their prescribed medication over 3 months. Those who were adherent and fully adherent fluctuated over the 13 weeks of intervention but ended week 13 approximately within 10% of adherence in week 1. a total of 189 alerts were triggered (all led to nurse phone calls mean 17.2 calls per month) 53 of these for missed medication, reminder for severe AE. End of study surveys were completed by 85 participants.<br><br>All patients reported BETA-Text was at least somewhat easy to use; 73% of respondents reported it helped them take their medication either very much or

	with severe symptoms (any type) Tamoxifen vs. AI vs. all: 14 vs. 24 ($p = 1$) vs. 38; Number of patients with severe hot flashes Tamoxifen vs. AI vs. all: 10 vs. 9 ($p = 0.1$) vs. 19; Number of patients with severe joint aches/pains Tamoxifen vs. AI vs. all: 3 vs. 12 ($p = 0.1$) vs. 15; Number of patients with severe vaginal symptoms Tamoxifen vs. AI vs. all: 3 vs. 2 ($p = 0.3$) vs. 5; Number of patients with severe other symptoms Tamoxifen vs. AI vs. all: 5 vs. 16 ($p = 0.2$) vs. 2		quite a lot. Over 70% reported no financial impact. Average of 10 min/week was spent using the application.4.7% felt it took up too much time. 3 patients reported incurring text-messaging fees. 69% reported they would want to continue receiving texts after the 3 months. All respondents reported missing 6 or fewer doses (less than 20%) in the previous month however adherence (measured by text response) was noted to be less the 80% in 10 of these patients. One patient reported a barrier in taking medication as she took to many medications in general.<br><br>Number of office visits I vs. C: 114 vs. 101, $p = 0.3$; Number of patient-initiated phone calls in I vs. C: 32 vs. 38, $p = 0.8$
Rico, 2017 [39]			All adherent patients reported having understood the content of the messages without difficulty executing them.<br><br>Patient over 60 years old did not adhere to the text messages. Her reason was being hospitalized after starting the treatment however she reported having read all the messages (this individual was not included in sample size).<br><br>All patients considered the text messages received helped them cope with treatment, felt these messages benefitted them. When asked why they helped: patients reported they felt more confident in treatment, felt supported and encouraged and that messages facilitated self-care, other patients reported they received new info about self-care and messages helped them to

		take better care of themselves. Some reported that they received certain orientations solely via text messages, some reported help psychologically via text messages. n = 13 reported being very satisfied n = 1 reported being satisfied, n = 14 adherent patients reported that they would recommend texts to other patients.
Sawicki, 2019 [40]		40% response rate to texts requiring a response, 5% patients requested consultation with pharmacist feature. The dasatinib group was the only one to achieve statistically significant different MPR in I vs. C (10.5% difference, $p = 0.01$). Optimal adherence was 53.4% in the clinical messaging patients and 43.7% in the control group (difference 9.7%, $p = 0.02$). Clinical messaging patients were 22% more likely to be optimally adherent. First fill drop-off rate in I vs. C: 4.7% vs. 10.0% (5.4% difference, $p = 0.02$). Average gap days for the intervention group and control groups were similar. Persistency was similar between first and second-line drugs; only 41% of patients remaining on therapy after 12 months. Intervention group patients were more likely to persist on first-line therapy (HR 1.2, 95% CI 0.8–1.7). The effect was similar with second-line therapies (HR 1.8, 95% CI 0.8–4.4).<br><br>All Therapies: Medication Possession Ratio (MPR) in I vs. C: 73.9 vs. 66.3, $p = 0.01$; Number of patients optimally adherent in I vs. C: 149 vs. 122, $p = 0.02$; Mean length of therapy (days) in I vs. C: 274.9 vs. 242.9, $p = 0.01$; Number of first fill drop-offs in I vs. C: 13 vs. 28, $p = 0.02$; Number of average gap days in I vs. C: 8 vs. 7.8 $p = 0.91$<br><br>First line therapies: MPR in I vs. C: 75.5 vs. 67.6, $p = 0.01$; Number of patients optimally adherent in I vs. C: 152 vs. 126, $p = 0.04$; Mean length of therapy (days) in I

	vs. C: 279.5 vs. 245.3, $p = 0.01$; Number of average gap days in I vs. C: C 7.8 vs. 7.5, $p = 0.9$; Number of first fill drop-offs in I vs. C: 11 vs. 27, $p = 0.01$<br><br>Imatinib MPR in I vs. C: 73.9 vs. 69.4, $p = 0.28$; Dasatinib MPR in I vs. C: 76 vs. 65.5, $p = 0.01$; Nilotinib MPR in I vs. C: 65.9 vs. 60.5, $p = 0.5$; Bosutinib MPR in I vs. C: 60.4 vs. 46.9, $p = 0.5$
Tan, 2019 [41]	Minor appointment tardiness (15–60 mins) was significantly more likely among those with NSMS (OR 1.25, 95% CI 1.13–1.38, $p < 0.0001$). It was also significantly associated with being in the first 3 fractions ($p < 0.0001$), having an earlier appointment time ($p < 0.0001$), being younger ($p = 0.0352$), and being male ($p = 0.0003$).<br><br>Major appointment tardiness (>60 mins) was significantly more likely among those with NSMS (OR 1.56, 95% CI 1.34–1.82, $p < 0.0001$). It was also significantly associated with being in the first 3 fractions ($p < 0.0001$), having an earlier appointment time ($p < 0.0001$), being younger ($p = 0.0024$), and being male ($p < 0.0001$).<br><br>No-show to appointments rate was significantly more likely among those with NSMS (6.77, 95% CI 5.45-8.41, $p < 0.0001$). It was also significantly associated with being in the first 3 fractions ($p < 0.0001$), having a later appointment time ($p < 0.0001$), having a further distance to treatment ($p = 0.0067$), and being younger ($p < 0.0001$).<br><br>There were significant differences in who opted into SMS by age, sex, distance to treatment, and time of appointment.

Wells, 2020 [42]		Depression (PHQ-9) reduced by 2.3 points (95% CI: 0.76–3.89) $p = 0.004$ more amongst smart-messaging users. Mean -4.7 SD 4.6 $p < 0.001$ from baseline to one-month post-treatment in the I group.<br><br>Anxiety (GAD-7) was not significantly different between I and C (data not reported). Mean-4.6 SD 5.7 $p < 0.001$ from baseline to one-month follow up in I group.	MBCT completion in I vs. C: 87% vs. 38%, $p = 0.007$, adjusted OR 7.8, 95% CI 1.8, 34.6<br><br>Of the 13 patients who used smart messaging and were interviewed found smart messages to be a prompt and reminder, some also found it motivating or drew patients back to mindfulness, second theme of personal connection was found (i.e., "someone is thinking about me") even when patient knew the message wasn't personally sent.<br><br>Two patients explained opting out due to lack confidence in mobile phones.<br>Smart messaging requested further (not being able to understand messages, feel like they should understand mobile phones but are unable to) messages 14 times on average during MBCT (SD 16, range 0–50)

Abbreviations: Adjuvant endocrine therapy (AET), Adverse Event (AE) Antihypertensive (AHT), Advanced Symptom Management System Management System (ASyMS), Body Mass Index (BMI), Center for Epidemiological Studies Depression Scale (CES-D), Control group (C), Confidence interval (CI), Electrochemiluminescence immunoassay (ECLIA), Edmonton Symptom Assessment System (ESAS), Effect size (ES), Functional Assessment of Cancer Therapy-Lung (FACT-L), Generalized Anxiety Disorder-7 (GAD-7), Hour (h), Hazard ratio (HR), Hormone receptor positive (HR-positive), Intervention group (I), Intention to treat (ITT), Interquartile range (IQR), Medication Adherence Self-Efficacy Scale-Revised (MAES-R), Mean difference (MD), Minute (min), Mindfulness-based cognitive therapy (MBCT), Metabolic equivalent (MET), Multidimensional Fatigue Inventory (MFI-20), Medication Possession Ratio (MPR), multivariate (MV), Moderate to vigorous physical activity (MVPA), No SMS (NSMS), Oral Anticancer (OA), Physical Activity (PA), Primary Cancer Provider (PCP), Patient Health Questionnaire-4 (PHQ-4), Patient-Reported Outcomes Measurement Information System (PROMIS), Quality of Life Questionnaire (QLQ-30), Randomized Control Trial (RCT), Standard deviation (SD), Short Form (SF), Simplified Medication Adherence Questionnaire (SMAQ), Simple Message Service (SMS), State-Trait Anxiety Inventory (STAI-Y), Strategies Used by People to Promote Health (SUPPH29), Tyrosine Kinase Inhibitor (TKI), Univariate (UV). Note: Outcome sample sizes are equal to sample sizes at baseline unless otherwise specified.

Table S4. Cochrane Risk of Bias table for the randomized controlled trials (n = 9). A score of 0 represents high risk of bias, 1 represents unclear risk of bias, and 2 represents low risk of bias.

Cochrane Risk of Bias Tool Criteria								
**Study ID**	**Selection Bias**		**Performance Bias**	**Detection Bias**	**Attrition Bias**	**Reporting Bias**	**Other Bias**	**Total**
	Random Sequence Generation	Allocation Concealment	Blinding of Participants & Personnel	Blinding of Outcome Assessment	Incomplete Outcome Data	Selective Reporting	Other Sources of Bias	
Casillas 2019 [26]	2	2	0	0	2	2	2	10
Gomersall 2019 [27]	2	2	0	0	2	2	2	10
Haggerty 2017 [28]	2	2	0	0	0	2	2	8
Hershman 2020 [29]	2	1	0	2	2	2	2	11
Rico 2020 [30]	1	1	0	0	1	2	2	7
Spoelstra 2016 [34]	2	2	0	0	2	2	2	10
Spoelstra 2015 [33]	2	2	0	0	1	2	2	9
Tan 2020 [31]	1	1	0	0	2	2	2	8
Villaron 2018 [32]	1	1	0	0	0	2	2	6

Table S5. Risk Of Bias In Non-randomised Studies - of Interventions (ROBINS-I) table for the non-randomized interventional/observational studies (*n* = 9).

**ROBINS-I Risk of Bias Criteria.**								
**Study ID**	**Confounding**	**Selection of Participants**	**Classification of Interventions**	**Deviations from Intended Interventions**	**Missing Data**	**Measurement of Outcomes**	**Selection of the Reported Results**	**Overall**
Bade 2018 [35]	Low	Serious	Serious	Low	Serious	Moderate	Serious	Serious
Chow 2019 [36]	Low	Low	Low	No Information	No Information	Moderate	Low	No Information
Krok-Schoen 2019 [11]	Low	Low	Low	Moderate	Serious	Moderate	Low	Serious
Maguire 2015 [37]	Low	No Information	Low	Low	Serious	Moderate	Serious	Serious
Mougalian 2017 [38]	Low	Serious	Low	Low	Low	Moderate	Low	Serious
Rico 2017 [39]	Low	Low	Low	Low	Moderate	Moderate	Low	Moderate
Sawicki 2019 [40]	No Information	No Information	Serious	No Information	No Information	Moderate	Low	No Information
Tan 2019 [41]	No Information	Low	Serious	Low	No Information	Low	Low	Serious
Wells 2020 [42]	Low	No Information	Serious	No Information	Moderate	Moderate	Low	Serious
